# Physiological Age- and Sex-Related Profiles for Local (Aortic) and Regional (Carotid-Femoral, Carotid-Radial) Pulse Wave Velocity and Center-to-Periphery Stiffness Gradient, with and without Blood Pressure Adjustments: Reference Intervals and Agreement between Methods in Healthy Subjects (3–84 Years)

**DOI:** 10.3390/jcdd8010003

**Published:** 2021-01-12

**Authors:** Daniel Bia, Yanina Zócalo

**Affiliations:** Departamento de Fisiología, Facultad de Medicina, Centro Universitario de Investigación, Innovación y Diagnóstico Arterial (CUiiDARTE), Universidad de la República, General Flores 2125, 11800 Montevideo, Uruguay

**Keywords:** adolescents, adults, arterial stiffness, blood pressure, children, pulse wave velocity

## Abstract

In addition to being a marker of cardiovascular (CV) aging, aortic stiffening has been shown to be independently associated with increased CV risk (directly and/or indirectly due to stiffness-gradient attenuation). Arterial stiffness determines the rate at which the pulse pressure wave propagates (i.e., pulse wave velocity, PWV). Thus, propagated PWV (i.e., the distance between pressure-wave recording sites divided by the pulse transit time) was proposed as an arterial stiffness index. Presently, aortic PWV is considered a gold-standard for non-invasive stiffness evaluation. The limitations ascribed to PWV have hampered its use in clinical practice. To overcome the limitations, different approaches and parameters have been proposed (e.g., local PWV obtained by wave separation and pulse wave analysis). In turn, it has been proposed to determine PWV considering blood pressure (BP) levels (β-PWV), so as to evaluate intrinsic arterial stiffness. It is unknown whether the different approaches used to assess PWV or β-PWV are equivalent and there are few data regarding age- and sex-related reference intervals (RIs) for regional and local PWV, β-PWV and PWV ratio. Aims: (1) to evaluate agreement between data from different stiffness indexes, (2) to determine the need for sex-specific RIs, and (3) to define RIs for PWV, β-PWV and PWV ratio in a cohort of healthy children, adolescents and adults. Methods: 3619 subjects (3–90 y) were included, 1289 were healthy and non-exposed to CV risk factors. Carotid-femoral (cfPWV) and carotid-radial (crPWV) PWV were measured (SphygmoCor System (SCOR)) and PWV ratio (cfPWV/crPWV) was quantified. Local aortic PWV was obtained directly from carotid waves (aoPWV-Carotid; SCOR) and indirectly (generalized transfer function use) from radial (aoPWV-Radial; SCOR) and brachial (aoPWV-Brachial; Mobil-O-Graph system (MOG)) recordings. β-PWV was assessed by means of cardio-ankle brachial (CAVI) and BP-corrected CAVI (CAVIo) indexes. Analyses were done before and after adjustment for BP. Data agreement was analyzed (correlation, Bland-Altman). Mean and standard deviation (age- and sex-related) equations were obtained for PWV parameters (regression methods based on fractional polynomials). Results: The methods and parameters used to assess aortic stiffness showed different association levels. Stiffness data were not equivalent but showed systematic and proportional errors. The need for sex-specific RIs depended on the parameter and/or age considered. RIs were defined for all the studied parameters. The study provides the largest data set related to agreement and RIs for stiffness parameters obtained in a single population.

## 1. Introduction

The arterial wall thickens and stiffens with aging. Such aging-related structural and functional changes are not distributed homogeneously but predominate in the aorta and central elastic arteries [1,2]. On the other hand, the mechanisms linking arterial stiffness and vascular disease (e.g., atherosclerotic) are not completely understood, in addition to being a marker of cardiovascular (CV) aging, aortic stiffening has been shown to be independently associated with increased CV risk and morbi-mortality [3]. This accounts for the interest in assessing aortic stiffness in the clinical practice.

Arterial stiffness determines the rate at which the pulse pressure wave propagates, that is, the pulse wave velocity (PWV). Thus, propagated PWV (obtained as the distance traveled by the pulse wave divided by the transit time) was proposed as an arterial stiffness index. Furthermore, up to now, PWV (in particular the carotid-femoral PWV (cfPWV)) is considered a gold standard for non-invasive arterial stiffness evaluation [1,4]. Although PWV is the most validated method to evaluate arterial stiffness in a non-invasive way, the limitations ascribed to PWV have hampered its use in clinical practice, the development in the field and the recognition of PWV as an independent marker of the vascular status [1]. About this, PWV measurement requires some skills, and pulse wave recording is difficult in some patients. On the other hand, PWV is inherently dependent on blood pressure (BP), which, if not considered, would lead to an inaccurate interpretation of PWV data [3,5,6]. To overcome such limitations, different approaches and parameters have been proposed. Then, methods relying on pulse-wave analysis and wave separation analysis (PWA and WSA, respectively) have been developed to non-invasively assess (local) PWV, directly from measured waves (e.g., carotid) or indirectly, from central waves mathematically-derived using general transfer functions (GTF) applied to peripheral waves (e.g., brachial, radial). In turn, the use of cuff-based oscillometric devices that give an estimated (local) PWV based on PWA and/or WSA requires less expertise, makes the process less operator-dependent and enables ambulatory measurements [7]. Finally, the stiffness index β and cardio-ankle vascular index (CAVI) would have the advantage of considering the relationship between stiffness and BP. Hence, they were proposed to evaluate intrinsic wall stiffness [6].

The detrimental impact of arterial stiffness on the CV system would be mediated by a direct effect of the increased stiffness and/or by the attenuation of the center-to-periphery stiffness gradient (e.g., evaluated by the PWV ratio). In this regard, the age-related reduction in the stiffness gradient is associated with adverse clinical outcomes [8]. Then, analyzing the PWV ratio would add to and complement data obtained from PWV.

Currently, different devices, approaches and parameters are used to non-invasively assess arterial stiffness. It is unknown whether the different methods used to quantify PWV provide equivalent information and/or if it is possible to define equations to convert data obtained with a given approach into data corresponding to another one. Additionally, there is little information regarding age and/or sex-related reference intervals (RIs) for regional and local PWV, β-PWV (or CAVI) and PWV ratio obtained at the same time in a large healthy population including children, adolescents and adults.

This work’s aims were: (1) to evaluate agreement between PWV data obtained with different approaches, (2) to determine the need for sex-specific RIs, and (3) to define RIs for PWV, β-PWV and PWV ratio in a cohort of healthy children, adolescents and adults from South America.

## 2. Materials and Methods

### 2.1. Study Population

The study was carried out in the context of the Centro Universitario de Investigación, Innovación y Diagnóstico Arterial (CUiiDARTE) project [9,10,11,12,13], a population-based study developed in Uruguay. In this work, we considered data from 3619 subjects included in the CUiiDARTE Database. This includes data on demographic and anthropometric variables, exposure to CV risk factors (CRFs), personal and family history of CV disease and data on structural and functional CV parameters non-invasively obtained, mainly from community-based projects [9,10,11].

To determine RIs (age- and sex-specific normative tables) for arterial stiffness parameters, we selected a healthysub-population that included children, adolescents and adults who did not meet any of the following criteria: (i) history of CV disease (defined as presence of cerebrovascular, coronary heart, valvular or peripheral arterial disease, impaired cardiac ejection fraction and/or left ventricular hypertrophy); (ii) use of BP-, lipid- and/or glucose-lowering drugs; (iii) arterial hypertension (≥18 y: brachial systolic blood pressure (bSBP) ≥ 140 mmHg and/or brachial diastolic blood pressure (bDBP) ≥ 90 mmHg); <18 y: bSBP and bDBP < 95th percentile for sex, age and body height (BH)); (iv) current smoking; (v) diabetes (defined as self-reported and/or fasting plasma glucose ≥ 126 mg/dL (if available)); (vi) dyslipidemia (defined as self-reported, total cholesterol ≥ 240 mg/dL or HDL cholesterol < 40 mg/dL (if available)); (vii) obesity (≥18 y: body mass index (BMI) ≥ 30 kg/m^2^; <18 y: z-BMI ≥ 2.0). The cut-off values used to define the healthy sub-population were chosen, whenever possible, to be similar to those used to indicate increased risk in clinical guidelines (or risk algorithms) [14,15,16,17], and to enable optimal comparison with data from other groups (e.g., the European Reference Values for Arterial Measurements Collaboration Group) [18,19,20,21]. Additionally, none of the subjects in the RIs group had congenital, chronic or infectious diseases and cardiac rhythm other than sinus rhythm. The resulting sub-population included 1289 individuals. All procedures are in agreement with the Declaration of Helsinki (1975 and reviewed in 1983) and the study protocol was approved by the Institution’s Ethics Committee. In adults, written informed consent was obtained prior to the evaluation. In children and adolescents (<18 y), parents’ written consent and children’s assent were obtained before the study. The procedures followed to obtain data used in this work are described below.

### 2.2. Anthropometric and Clinical Evaluation

A brief clinical interview, together with the anthropometric evaluation enabled us to assess CRFs exposure, defined according to the criteria (cut-off points) described above. A family history of CV disease was defined by the presence of first-degree (for all the subjects) and/or second-degree (for subjects ≤18 y) relatives with early (<55 y in males; <65 y in females) CV disease (see above). Bodyweight (BW) and BH were measured with the participants wearing light clothing and no shoes. Standing BH was measured using a portable stadiometer and recorded to the nearest 0.1 cm. BW was measured with an electronic scale (841/843, Seca Inc., Hamburg, Germany; model HBF-514C, Omron Inc., Chicago, IL, USA) and recorded to the nearest 0.1 kg. BMI was calculated as BW-to-squared BH ratio. In children and adolescents, z-scores for the BMI were calculated using the World Health Organization software (Anthro-v.3.2.2; Anthro-Plus-v.1.0.4) [9].

### 2.3. Cardiovascular Evaluation

Participants were asked to avoid exercise, tobacco, alcohol, caffeine and food-intake four hours before the evaluation. All hemodynamic measurements were performed in a temperature-controlled environment (21–23 °C), with the subject in supine position and after resting for at least 10–15 min, which enabled reaching steady hemodynamic conditions. Using a validated oscillometric device (HEM-433INT; Omron Healthcare Inc., Lake Forest, IL, USA), heart rate (HR), bSBP and bDBP were recorded simultaneously and/or immediately before or after each non-invasive tonometric (radial, femoral, carotid) and brachial oscillometry record. Then, brachial pulse pressure (bPP; bPP = bSBP − bDBP) and bMBP (bMBP = bDBP + bPP/3) were obtained.

CV evaluation in the CUiiDARTE project includes assessing: (i) peripheral (brachial, radial, ankle) and central (aortic, carotid) BP levels; central (aortic, carotid) PWA and WSA-derived parameters (e.g., augmentation index, forward and backward pressure components), (ii) carotid, femoral and brachial beat-to-beat diameter waves and intima-media thickness, (iii) brachial artery reactivity (e.g., flow-mediated dilation; low flow-mediated constriction), (iv) carotid, femoral and brachial doppler-derived blood velocity profiles and resistive/pulsatile indexes, (v) ankle-brachial index, (vi) screening for carotid and femoral atherosclerotic plaques presence, (vii) carotid, femoral and brachial local stiffness (e.g., distensibility, elastic modulus), (viii) systemic hemodynamic evaluation (e.g., systemic vascular resistances, cardiac output and index quantified from brachial pulse contour analysis and/or cardiography impedance, (ix) regional stiffness (cfPWV, crPWV) [9,10,11,12,13,22]. In this work, we focused on regional and local PWV data.

### 2.4. Carotid and Femoral Artery Ultrasound

Left and right common (CCA), internal and external carotid arteries and common femoral (CFA) arteries were examined (B-Mode and Doppler ultrasound, 7–13 MHz, linear transducer, M-Turbo, SonoSite Inc., Bothell, WA, USA) [22]. Transverse and longitudinal arterial views were obtained to assess the presence of atherosclerotic plaques. Near and far walls were analyzed, and images were obtained from anterior, lateral, and posterior angles. An atherosclerotic plaque was defined as: focal wall thickening at least 50% greater than the adjacent segment, focal thickening protruding into the lumen at least 0.5 mm or an intima-media thickness (IMT) ≥ 1.5 mm [23].

### 2.5. Regional PWV (cfPWV and crPWV) and Pulse Wave Velocity Ratio (PWV Ratio)

**Figure 1:**
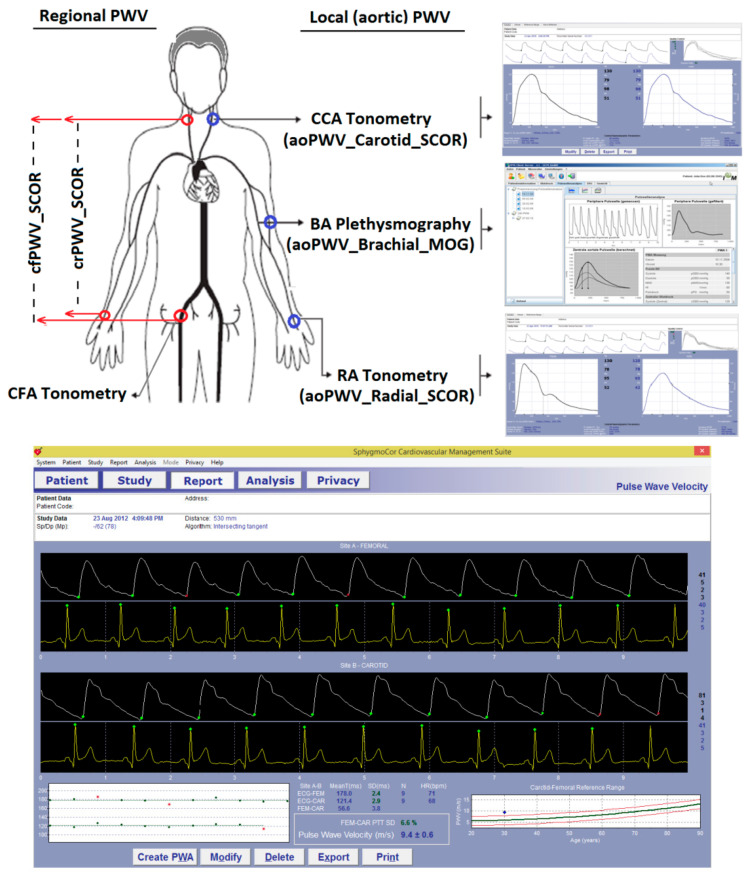
**Top**: Diagram showing arterial recording sites and pulse waves, measured or obtained to calculate local PWV. Software screens (SCOR and MOG) for carotid and radial applanation tonometry (SCOR) and for brachial oscillometry/plethysmography (MOG) records are shown. **Bottom**: software screen during cfPWV assessment showing the peripheral (femoral) and central (carotid) waves records and the resulting cfPWV (9.4 m/s).

Carotid-femoral (aortic; cfPWV) and upper arm (crPWV) regional stiffness were assessed (applanation tonometry, SphygmoCor-CvMS, AtCor-Medical, Sidney, Australia) (Figure 1). PWV values depend on the algorithm used for detecting the so-called “foot of the wave” and on the distance (path-length) considered [24,25,26,27]. In this work, the intersecting tangent algorithm was used to detect the wave-foot [24,25,26,27]. The path length can be the direct distance between the recording sites (e.g., carotid and femoral), or the distance obtained by subtracting the distance between the proximal recording site (e.g., carotid) and the sternal notch from the distance between the sternal notch and the peripheral recording site (e.g., femoral) [24,25,26,27]. Following international recommendations, for cfPWV, we used the direct distance multiplied by 0.8, which enabled us to obtain the real cfPWV. In turn, we considered the subtracted distance for crPWV quantification [24,25,26,27]. cfPWV and crPWV values were obtained from the median of three measurements (random order). PWV Ratio, a marker of center-periphery stiffness gradient was quantified as cfPWV/crPWV [28,29].

### 2.6. Local PWV: aoPWV_Radial_SCOR, aoPWV_Carotid_SCOR and aoPWV_Brachial_MOG

Central BP levels and waveforms were obtained (random order) using two commercially available devices: SphygmoCor-CvMS (SCOR; v.9, AtCor-Medical, Australia) and Mobil-O-Graph PWA-monitor system (MOG; I.E.M.-GmbH, Stolberg, Germany) (Figure 1). Both devices and systems enable PWA and WSA [12]. A detailed (step-by-step) explanation of the method used for WSA based on recorded (carotid wave, SCOR) and mathematicallyderived aortic wave (SCOR and MOG) was included in a previous work as Supplementary Material [12].

Using SCOR, central BP waves were derived from (i) radial (using a GTF) and (ii) carotid tonometric recordings (Figure 1). Only accurate waveforms on visual inspection and high-quality recordings (in-device quality index > 75%) were considered. Carotid artery pulse waves were assumed to be identical to the aortic ones (due to the proximity of the arterial sites) [12]. Thus, a GTF was not applied to obtain central waves from carotid records. In turn, brachial BP (bBP) levels and waveforms were obtained using the MOG (brachial cuff-based oscillometric/plethismographic device) [12]. The device determined central BP levels and waveforms from peripheral recordings using a validated GTF. Only high-quality records (index equal to 1 or 2) and satisfactory waves (visual inspection) were considered. Both devices (SCOR and MOG) quantify (local) PWV by proprietary algorithms (e.g., based on age, bSBP, pulse wave characteristics, transition time between WSA-derived forward and reflected components of the pulse wave) [30]. Data were named based on the recording site and device used: aoPWV_Radial_SCOR, aoPWV_Carotid_SCOR and aoPWV_Brachial_MOG (Figure 1).

### 2.7. CAVI and CAVIo Determination

Arterial stiffness (e.g., assessed by PWV) is influenced by BP levels during the examination, which, if not considered, could lead to inaccurate conclusions [5,6,31,32,33]. To overcome the problem of BP dependence, Shirai et al. proposed the use of the cardio-ankle vascular index (CAVI) [6], which “combines” the stiffness index β (commonly used to quantify local arterial stiffness) and the Bramwell–Hill equation, to obtain an index that can be calculated using arterial BP and PWV (unlike β which is calculated from arterial BP and diameter) [5,33]. CAVI was suggested to better reflect structural changes of the arterial wall (with independence of arterial distending BP). However, due to the use of bDBP instead of a fixed reference BP, both the stiffness index and CAVI theoretically have a residual dependence on BP. Additionally, CAVI exhibits BP dependence since it considers an estimated derivative of the pressure-diameter relationship. Recently, a BP-corrected CAVI (CAVIo) was proposed, which is suggested (theoretically) to represent a less BP-dependent stiffness index [5,33]. Although named cardio-ankle, the CAVI formula can equally well be applied to any PWV measurement [31,33]. CAVI or β-cfPWV was previously used in both children and adults [5,32]. In this work, CAVI and CAVIo were calculated as:

CAVI = (Ln(bSBP/bDBP))*((PWV^2^*2ρ)/bSBP − bDBP)) (1)

CAVIo = ((PWV^2^*2ρ)/bDBP) − Ln(bDBP/BPref) (2)

where Ln is natural logarithm, bSBP and bDBP are brachial systolic and diastolic BP, BPref is 100 mmHg, PWV is regional (cfPWV, crPWV) or local (aoPWV_Radial_SCOR, aoPWV_Carotid_SCOR, aoPWV_Brachial_MOG) PWV and ρ is blood mass density (assumed 1060 kg/m^3^) [5,33]. BP, PWV and ρ were entered into equation in Pa, m/s and kg/m^3^, respectively [5,33].

### 2.8. Arterial Stiffness Parameters

After completing the calculations, 18 stiffness-related parameters were obtained (Table 1).

### 2.9. Data Analysis

**Figure 2:**
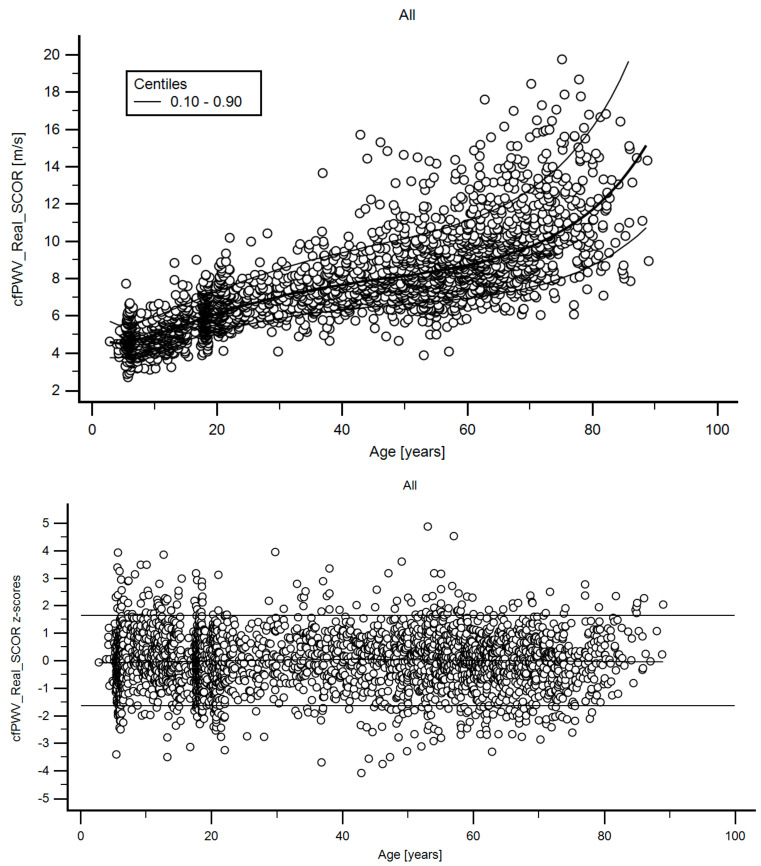
**Top**: Age-related cfPWV profile (10th, 50th, 90th percentiles). **Bottom**: z-score diagram used to verify model fit.

A step-wise analysis was performed. First, we assessed the association between age and arterial stiffness parameters, considering the whole population (*n* = 3169). Figure 2 shows the age-related profiles obtained for cfPWV. Similar data were obtained for the 18 PWV-related parameters.

Second, considering the inclusion and exclusion criteria, subjects to be included in the RIs group were identified (*n* = 1289) (Table 2 and Table 3). Additional complimentary data and sex distribution for the entire population (*n* = 3619), excluded subjects (*n* = 2330) and for the RIs group (*n* = 1289) are included in Supplementary Material 1 (Tables S1–S3).

**Figure 3:**
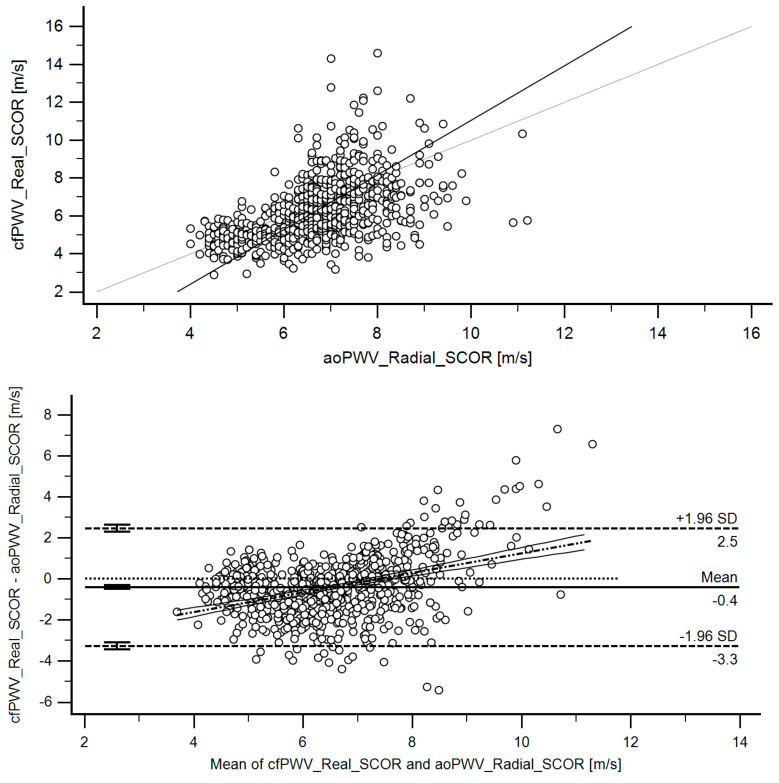
**Top**: Association (scatter diagram) between cfPWV_RealSCOR and aoPWV_Radial-SCOR for the RI Group. r: 0.4980 (95% C.I.: 0.4429–0.5493). *p* < 1.0 × 10^−14^. **Bottom**: Bland–Altman diagram. There were statistically significant mean (−0.41 m/s) and proportional errors. Table 5 (Part 1) shows quantitative data.

Third, working with the RIs group, we first analyzed the agreement between the methods used to assess aortic stiffness. Concordance correlation coefficients (CCC) (Table 4) and Bland–Altman tests were considered (Table 5). Figure 3 exemplifies CCC analysis and Bland–Altman graph obtained when comparing cfPWV_Real_SCOR and aoPWV_Radial_SCOR. Similar analyses were done for different parameters. The above enabled us to identify that the approaches and parameters considered did not provide similar data; they were not equivalent. Therefore, it was necessary to define the RIs for each parameter.

Fourth, we analyzed the association between carotid and/or femoral atherosclerotic plaque presence (asymptomatic subjects) and PWV data by means of point-biserial correlations without and with Bootstrapping (sample number = 1000; bias-corrected accelerated confidence intervals; simple sampling) (Table 6). In subjects non-exposed to CRFs (RIs Group), plaque presence was not associated with cfPWV, crPWV or aoPWV levels. Then, subjects with atherosclerotic plaques were not excluded from that group. As a result, our exclusion criteria agreed with those of the European Reference Values for Arterial Measurements Collaboration Group [18,19,20,21].

Fifth, we evaluated whether it was necessary to define sex-specific RIs using: (i) bivariate partial correlations (age-adjusted) and (ii) interaction analysis (Sex*Age) with theJohnson-Neyman significance regions definition (Table 7). Variables “y”, “x” and “w” (moderating variable) were assigned, respectively, to the stiffness parameter (e.g., cfPWV_Real_SCOR), sex and age. Then we identified: (i) parameters that would require RIs for males and females only from a certain age (cfPWV_Real_SCOR and crPWV_SCOR), (ii) parameters that would require sex-specific RIs for all ages (aoPWV_Brachial_MOG) and (iii) parameters that would not require sex-specific RIs (aoPWV_Radial_SCOR and aoPWV_Carotid_SCOR) (Table 7). In all cases, however, we decided to define RIs for all the subjects, as well as for females and males separately.

Finally, as a sixth step, age-related RIs were obtained. Age-related equations were obtained for mean values (MV) and standard deviation (SD). Then, we implemented parametric regression methods based on fractional polynomials (FPs), described by Royston and Wright [34], included in the European Reference Values for Arterial Measurements Collaboration Group methodological strategy [18,19,20,21] and already used by our group [13,35,36,37].

**Figure 4:**
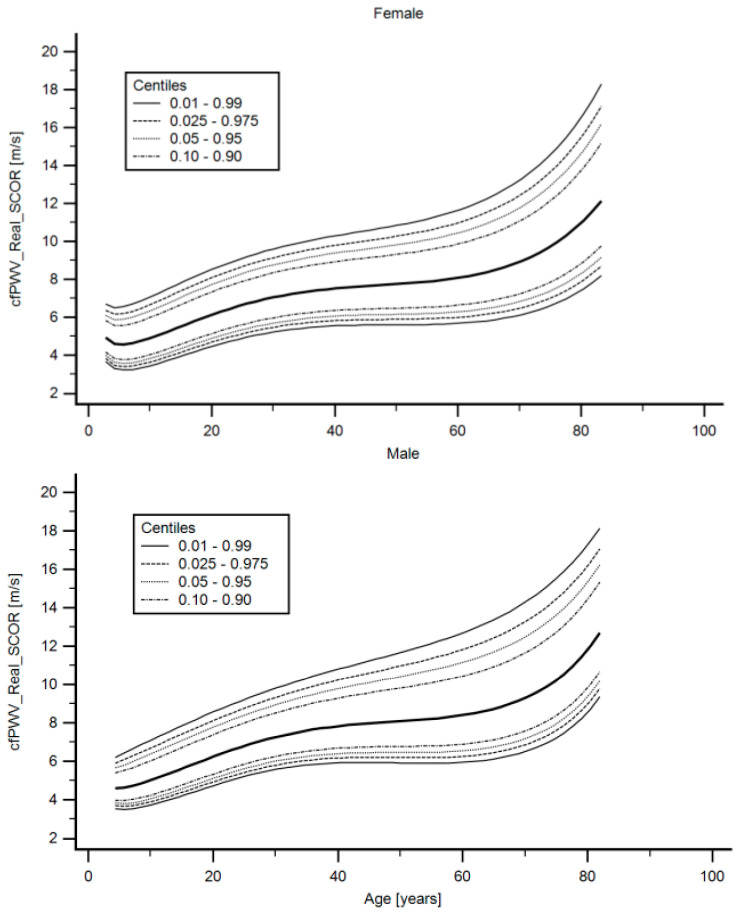
cfPWV_Real_SCOR age-related percentile curves for female (**top**) and males (**bottom**). Quantitative data are shown in Table 8 and Supplementary Material 1 and 2.

Briefly, fitting FPs age-specific MV and SD regression curves for the different variables (e.g., cfPWV_Real_SCOR) were defined using an iterative procedure (generalized least squares). Then, age-specific equations were obtained for the different parameters. For instance, the cfPWV_Real_SCOR MV equation would be cfPWV_Real_SCOR MV = a + b*Age^p^ + c*Age^q^ + …, where a, b, c, … are the coefficients, and p, q, … are the powers, with numbers selected from the set [−2, −1, −0.5, 0, 0.5, 1, 2, 3] estimated from the regression for mean cfPWV_Real_SCOR curve, and likewise from the regression for SD curve. Continuing the example, FPs with powers [1,2], that is, with p = 1 and q = 2, illustrate an equation with the form a + b*age + c*age^2^ [34]. Residuals were used to assess the model fit, which was deemed appropriate if the scores were normally distributed, with a mean of 0 and an SD of 1, randomly scattered above and below 0 when plotted against age. Best fitted curves, considering visual and mathematical criteria (Kurtosis and Skewness coefficients) were selected. Using the equations obtained for MV and SD (Supplementary Material 1, Table S4), age-specific percentiles were defined using the standard normal distribution (Z). The 1st, 2.5th, 5th, 10th, 25th, 50th, 75th, 90th, 95th, 97.5th and 99th percentiles were calculated, for example for cfPWV_Real_SCOR: mean cfPWV_Real_SCOR + Zp*SD, where Zp assumed the values −2.3263, −1.9599, −1.6448, −1.2815, −0.6755, 0, 0.6755, 1.2815, 1.6448, 1.9599 and 2.3263, respectively (Table 8, Table 9, Table 10, Table 11, Table 12 and Table 13). Applying this approach, RIs were quantified for the parameters listed in Table 1. Year by year RI data can be found in Supplementary Material 1 (Tables S5–S58). Supplementary Material 2 shows percentile curves (for all subjects, females and males) corresponding to the different stiffness parameters studied. Figure 4 illustratesthe age-related profiles obtained for cfPWV_Real_SCOR. Similar data were obtained for the remaining parameters (Supplementary Material 2).

The minimum sample size required was 377 [38]. Like in previous works and according to the central limit theorem, normal distribution was considered (taking into account Kurtosis and Skewness coefficients distribution and sample size > 30) [39]. Data analysis was done using MedCalc-Statistical Software (version 18.5, MedCalc Inc., Ostend, Belgium) and IBM-SPSS software (SPSS Inc., Chicago, IL, USA). PROCESS version 3.5 (SPSS extension) was used for moderation (interaction) analysis [40]. Evans’s Empirical Classifications of Interpreting Correlation Strength by Using r was applied: r < 0.20, very weak; r: 0.20–0.39, weak; r: 0.40–0.59, moderate; r: 0.60–0.79, strong; r ≥ 0.80, very strong [30,41]. *p* < 0.05 was considered statistically significant.

## 3. Results

### 3.1. Subjects’ Characteristics

Table 2 and Table 3 show subjects’ demographic, clinical, anthropometric and CV data. Note the wide age-range considered (in the population and the RIs group) (Table 2). As shown in Table 3, each arterial stiffness determination had a corresponding BP recording.

### 3.2. Association between Arterial Stiffness Parameters Obtained with Different Devices and/or Algorithms

Simple bivariate and concordance correlation data are shown in Table 4. Note the differences in Pearson coefficients (r) and CCC obtained when analyzing the cfPWV_Real_SCOR correlation with aoPWV data, obtained with other approaches (aoPWV_Radial_SCOR: r = 0.498, CCC = 0.448; aoPWV_Carotid_SCOR: r = 0.3145, CCC = 0.271 and aoPWV_Brachial_MOG: r = 0.7336, CCC = 0.614). Looking at the findings, it could be said: (i) although the positive associations were significant, they were weak or moderate, (ii) association levels varied depending on the devices and parameters compared, and (iii) statistical agreement (equivalence) levels were low (Table 4).

Likewise, regardless of whether it was considered aoPWV, CAVI or CAVIo, (i) the levels of association were always significant (except for the association between CAVI and CAVIo for aoPWV_Carotid_SCOR and aoPWV_Brachial_MOG), and (ii) in all cases, CCC showed low (or even non-existent) levels of agreement between data (Table 4). CCC values were always <0.617.

### 3.3. Agreement between Stiffness Parameters Obtained with Different Devices and/or Algorithms

Table 5 shows the Bland–Altman analyses carried out to determine the levels of equivalence between data obtained with different devices and/or algorithms. cfPWV_Real_SCOR and aoPWV data showed: (i) significant systematic differences (errors), whose mean values were −0.41 m/s, −0.14 m/s and 0.81 m/s, and (ii) proportional differences (errors), that is to say, differences that varied in magnitude depending on PWV values. With a negative intercept, the difference between cfPWV_Real_SCOR and any of the aoPWV indexes, had a positive slope, with the error crossing 0 at approximately 7 m/s. Thus, for low levels of stiffness, cfPWV_Real_SCOR showed lower values than aoPWV. As stiffness levels increased, the differences decreased, until the ratio was reversed and cfPWV_Real_SCOR reached higher values than aoPWV (Table 5). Likewise, methods used to quantify aoPWV showed significant systematic (mean: 0.28 m/s, 1.49 m/s and 1.38 m/s) and proportional errors (except aoPWV_Radial_SCOR and aoPWV_Carotid_SCOR) (Table 5).

CAVI data showed significant systematic differences. On the other hand, with the exception of CAVI_aoPWV_Radial_SCOR and CAVI_aoPWV_Carotid_SCOR, they also showed proportional differences. In turn, CAVIo data showed both, systematic and proportional errors (Table 5).

Jointly analyzing the findings, it could be said that the methods used to assess PWV, CAVI or CAVIo were not equivalent.

### 3.4. Arterial Stiffness and Atherosclerotic Plaques in Asymptomatic Subjects

There was no association between arterial stiffness (cfPWV, crPWV and aoPWV) and atherosclerotic plaque presence (Pearson coefficients ≤ 0.1, not significant) (Table 6).

### 3.5. Arterial Stiffness and Sex Differences

Table 6 shows that in a first analysis (simple bivariate associations), without taking into account adjustments and/or interactions with age, sex showed a slight negative association with cfPWV_Real_SCOR and aoPWV_Brachial_MOG (lower stiffness levels in females).

However, when age and sex interaction was considered, there were: (i) parameters that required sex-specific RIs from certain ages (cfPWV_Real_SCOR and crPWV_SCOR), (ii) parameters for which sex-specific RIs were necessary in all ages (aoPWV_Brachial_MOG) and (iii) parameters that did not require sex-specific RIs (aoPWV_Radial_SCOR and aoPWV_Carotid_SCOR) (Table 7, Part 1).

Females and males <19.67 y did not show significant differences in cfPWV_Real_SCOR (*p* > 0.05), while for older ages, cfPWV was gradually higher in males. About this: (i) at the age of ~23 y (23.15 y), cfPWV values in males were 0.1373 m/s higher than in females, (ii) at ~40 y (39.43 y), the difference was 0.2789 m/s, (iii) at the age of ~60 y (59.78 y), male values were 0.456 m/s higher than those in females whereas (iv) at ~80 y (80.13 y), the difference was 0.633 m/s. Similarly, from 29 years of age (28.9 y), the crPWV_SCOR levels were gradually higher in males than in females (0.220 m/s, 0.385 m/s, 0.659 m/s and 0.933 m/s at 28.9 y, 40.75 y, 60.50 y and 80.25 y, respectively) (Table 7, Part 2). On the other hand, aoPWV_Brachial_MOG was associated with sex, with the independence of age, whereas aoPWV_Radial_SCOR and aoPWV_Carotid_SCOR did not show an association with sex, nor an interaction between age and sex (Table 7, Part 3). In all cases, however, we decided to define RIs for all the subjects, as well as for females and males separately.

### 3.6. Age- and Sex-Related Reference Intervals

Table 8, Table 9, Table 10, Table 11, Table 12 and Table 13 show a summary (5 y intervals) of the reference values (p50th, p75th, p90th, p95th, p97.5th, p99th) for each stiffness parameter (its CAVI and CAVIo). Data for year-by-year intervals can be seen in Supplementary Material 1 (Tables S5–S58).

## 4. Discussion

### 4.1. Main Findings

**Figure 5:**
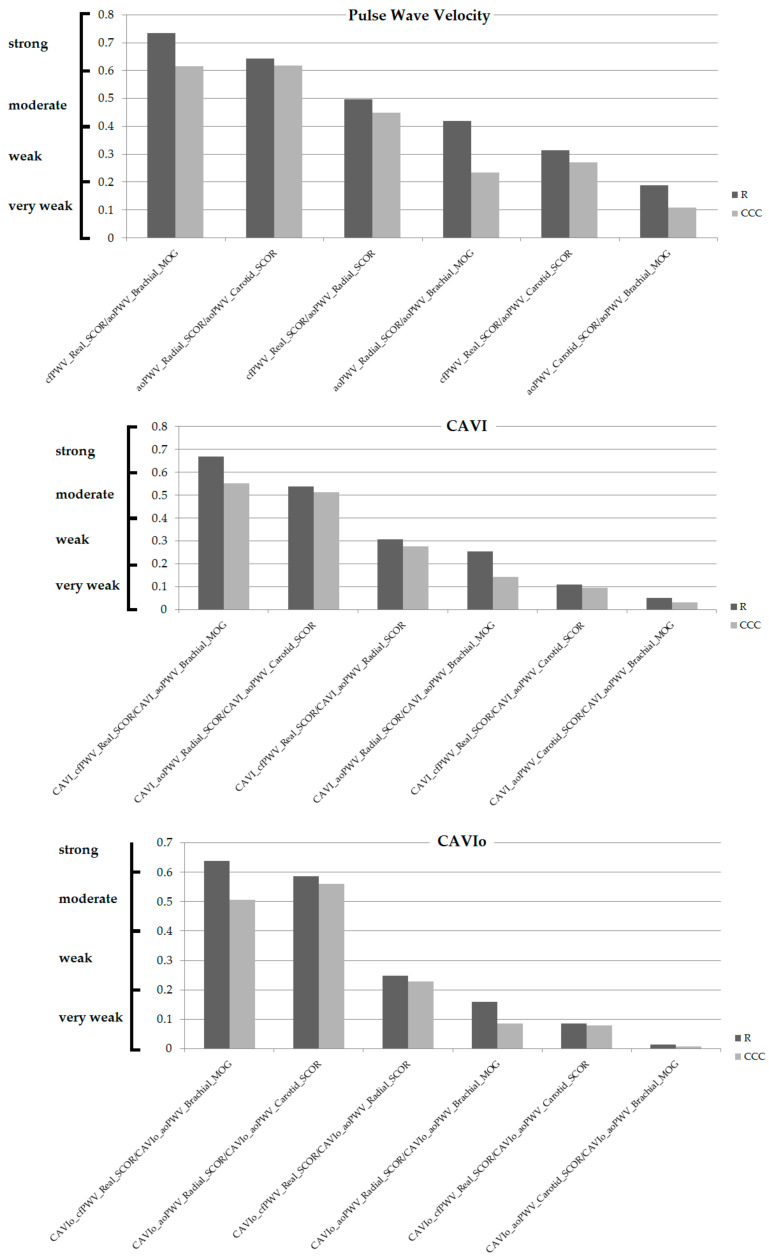
Simple (Pearson, r) and concordance correlation coefficients (CCCs), ordered from highest to lowest based on r value, for PWV (**top**), CAVI (**middle**) and CAVIo (**bottom**). Evans’s Empirical Classifications of Interpreting Correlation Strength by Using r was applied: (i) very weak: <0.20; (ii) weak: 0.20–0.39, (iii) moderate: 0.40–0.59, (iv) strong: 0.60–0.79, (v) very strong: r ≥ 0.80 [41].

The work’s main findings can be summarized as follows:

- First, for methods and parameters used to assess aortic stiffness (cfPWV_Real, aoPWV_Radial, aoPWV_Carotid and aoPWV_Brachial), their CAVI and CAVIo, showed different association levels (some non-significant). In no case was the association very strong (r ≥ 0.80) (Table 4, Figure 5).

For PWV, CAVI and CAVIo, the highest levels of association were obtained for cfPWV_Real_SCOR and aoPWV_Brachial_MOG (r = 0.73, r = 0.67 and r = 0.64, respectively), whereas the lowest levels were obtained for aoPWV_Carotid_SCOR and aoPWV_Brachial_MOG (r = 0.19, r = 0.05 and r = 0.01, respectively). Regardless of whether or not there was agreement (equivalence) on absolute levels, there were methods with no association, so it would not be possible to arrive at similar conclusions from similar trends in their variations (Table 4, Figure 5).

Recently, Salvi et al. obtained an r = 0.64 (*p* < 0.0001; mean error/SD: 0.40 ± 2.23 m/s) for the association between aoPWV_Brachial_MOG and cfPWV_Real_SCOR [30]. Similarly, when aoPWV_Brachial_MOG was considered, the levels of association with propagated cfPWV data were heterogeneous and in no case achieved “very strong” values: (i) Complior System (*n* = 100, r = 0.46; *p* < 0.0001; mean error/SD: 0.28 ± 2.94 m/s); (ii) PulsePen ETT System (*n* = 99, r = 0.62; *p* < 0.0001; mean error/SD: 1.17 ± 2.59 m/s); (iii) PulsePen ET System (*n* = 102, r = 0.61; *p* < 0.0001; mean error/SD: 1.00 ± 2.77 m/s) [30]. In agreement with our findings (but working with an *n* = 102) the authors found differences in the associations between methods used to assess aortic stiffness (in some cases, they did not reach statistical significance). On the contrary, in the work of Salvi et al., methods that evaluated propagated PWV (e.g., SphygmoCor, Complior, PulsePen ET, PulsePen ETT) showed Pearson Coefficients > 0.8 [30].

**Figure 6:**
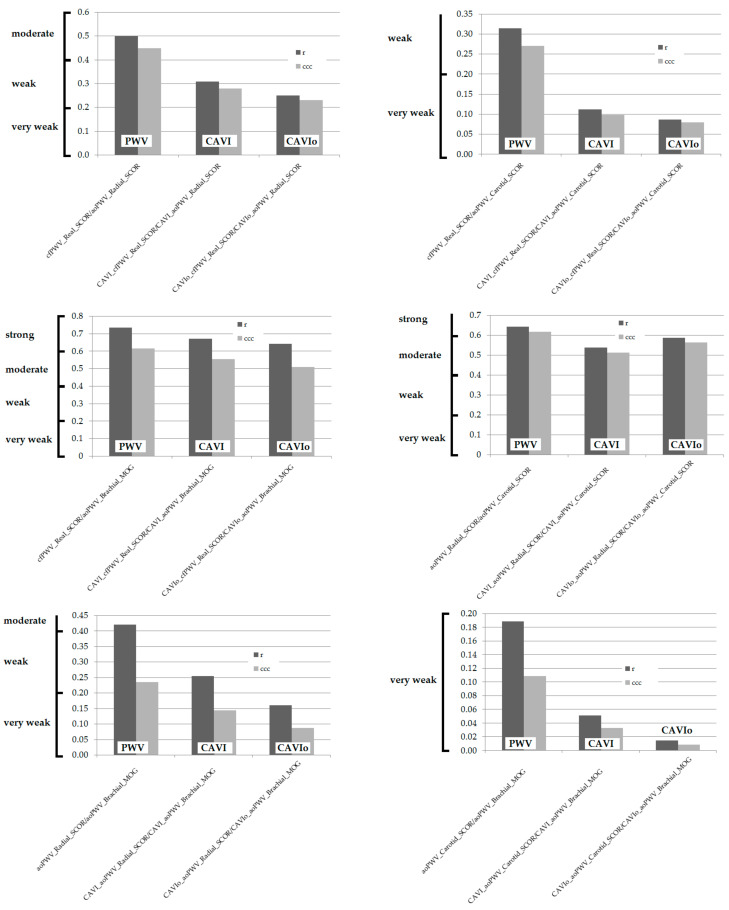
Simple (Pearson, r) and concordance correlation coefficients (CCCs) for PWV, CAVI and CAVIo, ordered for “pairs” of records. Evans’s Empirical Classifications of Interpreting Correlation Strength by Using r was applied: (i) very weak: <0.20; (ii) weak: 0.20–0.39, (iii) moderate: 0.40–0.59, (iv) strong: 0.60-–0.79, (v) very strong: r ≥ 0.80 [41].

On the other hand, when BP dependence was considered (CAVI and CAVIo), there was a reduction in the levels of association (Figure 6). In all cases, there was a gradual reduction in r, from the analysis of stiffness parameters (cfPWV and/or aoPWV), their transformation into CAVI and into corrected CAVI (CAVIo). The reduction in r led to changes in the categorization of the associations (e.g., moderate for aoPWV_Radial_SCOR/aoPWV_Brachial_MOG, very weak for CAVIo_aoPWV_Radial_SCOR/CAVIo_aoPWV_Brachial_MOG) (Figure 6). Therefore, considering (becoming independent of) BP levels at the time of arterial stiffness determination, resulted in a reduction in the associations between stiffness parameters, rather than in their “strengthening” due to the elimination of a factor, potentially different and determinant of the stiffness values.

**Figure 7:**
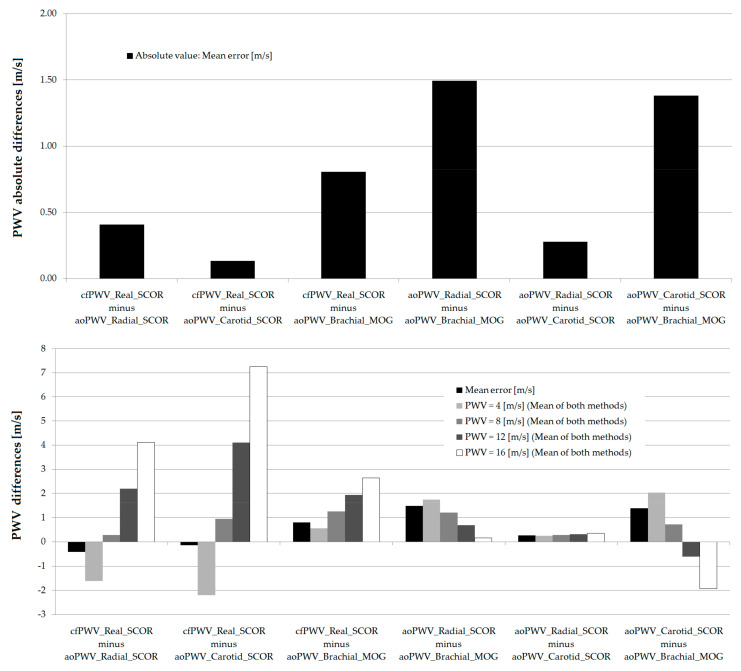
**Top**: Mean error (absolute value) for PWV data from different methods (Bland–Altman analysis). **Bottom**: Mean error and proportional differences for PWV equal to 4, 8, 12 and 16 m/s.

- Second, approaches and parameters used to assess aortic stiffness (cfPWV_Real, aoPWV_Radial, aoPWV_Carotid and aoPWV_Brachial) were not equivalent but showed systematic and proportional errors (Table 5, Figure 7).

Mean differences (absolute mean errors) were between 0.13 and 1.5 m/s (Table 5, Figure 7). In turn, when analyzing proportional errors: (i) three comparisons showed proportional differences, with a method always higher (or lower) than the other one, and (ii) three comparisons showed proportional differences in which the method with higher values varied depending on the stiffness level considered (Table 5, Figure 7).

Salvi et al. found a mean error equal to 0.40 ± 2.23 m/s (mean ± SD) when analyzing aoPWV_Brachial_MOG and cfPWV_Real_SCOR data. In addition, the authors found a negative proportional bias [30]. The findings agree with this work, in which a mean error equal to 0.81 m/s was observed for cfPWV_Real_SCOR and aoPWV_Brachial_MOG difference. In the work of Salvi et al., correlation (showing the equality line) and Bland–Altman graphs showed a trend that would enable the inference that as PWV increases, cfPWV_Real_SCOR would be gradually greater than aoPWV_Brachial_MOG [30]. Similarly, in this work, the differences between methods (cfPWV_Real_SCOR minus aoPWV_Brachail_MOG) increased at higher PWV values (Figure 7). For mean PWV values equal to 4, 8, 12 and 16 m/s, cfPWV_Real_SCOR was, respectively, 0.56, 1.26, 1.95 and 2.64 m/s higher than aoPWV_Brachial_MOG (Figure 7). As described in the above-mentioned work, at very low PWV values (e.g., the theoretical level close to the intercept), the relationship would be reversed. Although it is beyond this work’s scope, at least in theory the differences could be explained by the way aoPWV_Brachial_MOG is obtained. The algorithm used by the MOG device would provide PWV estimates, mainly calculated from age and bSBP data. Then, unlike propagated cfPWV measurements, aoPWV_Brachial_MOG could not show stiffness changes not explained by age and/or BP (e.g., explained by exposure to non-traditional CRFs). Related to what is stated above, Salvi et al. [30] went further and established that the Mobil-O-Graph should be considered an algorithm-based system, rather than an oscillometric cuff-based one. Indeed, they suggested the device would not provide measurements, nor estimations of aortic PWV, but would give the calculation of expected PWV values for a given age and bSBP [30]. In this context, it should be noted that in all cases, the maximum differences (absolute mean error) were found when comparing PWV data from MOG and SCOR (cfPWV_Real_SCOR, aoPWV_Radial_SCOR or aoPWV_Carotid_SCOR) (Figure 7).

PWV data showed low (the lowest) differences when aoPWV_Radial_SCOR and aoPWV_Carotid_SCOR were compared. Statistically significant mean and proportional errors were observed when they were compared to cfPWV_Real_SCOR (Table 5, Figure 7).

- Third, the need for sex-specific arterial stiffness RIs relied on the approach used to assess stiffness and/or on the age considered. In this respect, according to the measurement system, sex-specific RIs were not necessary, necessary regardless of age or only needed after a certain age (Table 7).

The relationship between sex and arterial stiffening is controversial and at least two issues are still discussed: (i) whether there are sex-related differences in mean arterial stiffness as a function of age, and (ii) whether there are sex-related differences in the rate of age-associated stiffness increase. In this work, we found that both issues may be closely related. The confusion or controversy regarding them could depend on the site of recording and/or on the approach (and device) used to assess arterial stiffness.

The aoPWV_Brachial_MOG values were systematically higher in males than in females (Table 7, Part C). At least in theory (in line with the aforementioned), the algorithm used to obtain aoPWV_Brachial_MOG, in which bSBP has a significant contribution, together with the higher bSBP levels (generally) found in men, could contribute to explaining the findings. In other words, sex-related differences in stiffness could be explained by the method used to calculate aoPWV with the MOG. Related to this, aoPWV_Carotid_SCOR and aoPWV_Radial_SCOR did not show sex-related differences, although for aoPWV_Radial_SCOR they were close to the statistical significance (*p* = 0.055). In addition, none of the variables showed a significant interaction between age and sex. Unfortunately, the internal algorithms that allow quantifying aoPWV by means of the different approaches are unknown, which does not allow adequate analysis of factors that could explain or be associated with the findings (e.g., lack of differences).

For propagated PWV (cPWV and crPWV), there were no significant sex-related differences in childhood, but thereafter these became gradually larger (higher stiffness in males than in females). This is in agreement with previous data published by our group regarding local carotid stiffness (carotid elastic modulus) in children and adolescents [42]. The findings also agree with results (for adults) obtained in the Anglo-Cardiff Collaboration Trial cohort, in which cfPWV was on average 2% lower in women than in men, without sex-related differences in the stiffening rate [43]. In this work, sex-related differences in cfPWV were observed earlier (a decade) than in crPWV.

As was discussed the need for RIs separated by sex varied depending on the approach used to assess arterial stiffness. In all cases, we opted for defining RIs for the population as well as for males and females separately.

- Fourth, this work’s main result is the definition of population-based reference values for PWV, CAVI and CAVIo indexes, obtained in the same subjects, with different non-invasive approaches. The definition of RIs is an important step when considering the introduction of PWV, CAVI and CAVIo indexes as a tool for the detection of subclinical target organ damage in the general population (Table 8, Table 9, Table 10, Table 11 and Table 12, Supplementary Material 1 (Tables S5–S55)). To our knowledge, this is the first time RIs are defined for the center-to-periphery arterial stiffness ratio (gradient) (Table 13, Supplementary Material 1 (Tables S56–S58)).

In biomechanical terms, while the impact of aortic stiffness on the myocardium is explained by increased cardiac afterload and reduced perfusion, peripheral target organ damage is best explained by the arterial stiffness gradient hypothesis [44]. As a result of the heterogeneity in vascular wall composition and geometry [45,46], arterial stiffness increases from central (e.g., ascending aorta) to peripheral (e.g., radial) arteries (center-to-periphery stiffness gradient). Central large elastic and peripheral medium-sized muscular conduit vessels, which are structurally and biomechanically different, would not be similarly affected by factors like aging, disease and drugs [47]. In this regard, the aging-related stiffness increase mainly occurs in the central elastic arteries. As the aorta stiffens, there is an attenuation and even a reversal of the stiffness gradient, resulting in a reduction in the attenuation of the forward pressure wave, and in an increased pulse pressure transmission to the microcirculation. This would lead to vascular myogenic response, endothelial dysfunction, hypoperfusion and organ damage [44,47,48]. Recent studies underlined the meaning of the stiffness gradient for both clinical conditions and outcomes [49,50,51]. It was proposed that the center-periphery PWV ratio would predict mortality better than cfPWV alone [47,48]. In this context, an issue that should be noted and addressed is the lack (so far) of: (i) data about age-related PWV ratio profiles, (ii) RIs (MV or SD equations) for PWV ratio that could, for example, allow assessing the meaning of deviations from expected levels in terms of cardiovascular status and prognosis.

In this work, RIs for stiffness parameters were obtained working with a large population of healthy subjects, selected taking into account inclusion and exclusion criteria previously used by our and other groups. Tables and Figures showing age-related (year by year) profiles for the different parameters (percentiles: 1, 2.5, 5, 25, 50, 75, 90, 95, 97.5 and 99th) are included in Supplementary Material 1 and 2.

**Figure 8:**
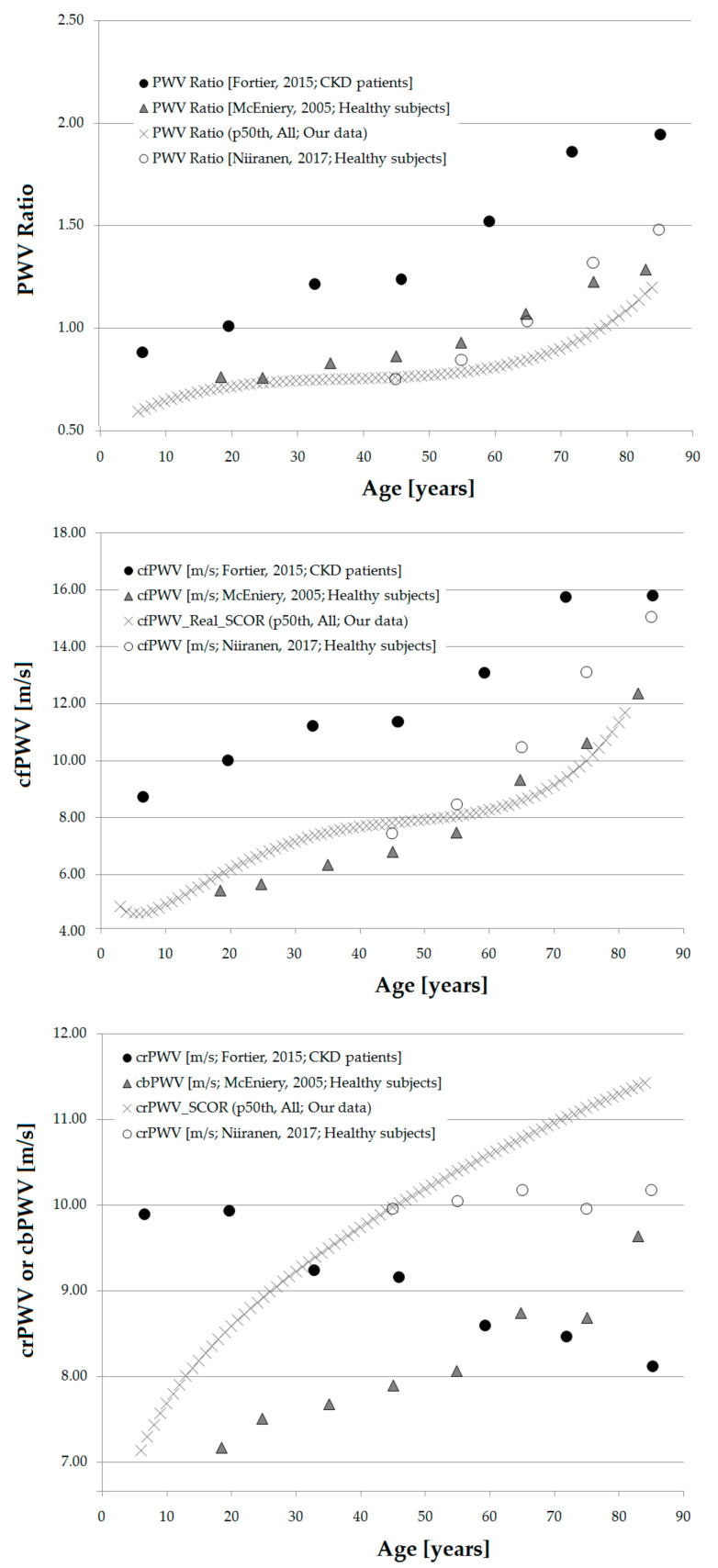
Comparative age-related profiles for PWV Ratio (**Top**), cfPWV (**Middle**) and crPWV or carotid-brachial PWV (cbPWV) (**bottom**).

Figure 8 shows RIs for cfPWV_Real_SCOR, crPWV_SCOR and PWV ratio (computed as cfPWV_Real_SCOR/crPWV_SCOR), obtained from our data, together with profiles published by other authors. Unfortunately, despite several groups having published cfPWV data, only a few have assessed crPWV, and even fewer have shown values as a function of age. Note the similarity between the PWV ratio data from this work and those obtained in healthy subjects by McEniery et al. [52] and Niiranen et al. [53], although in our work the PWV Ratio = 1 was obtained at older ages. McEniery et al. registered carotid-brachial PWV (cbPWV), whose values are expected to be lower than those of crPWV (forearm arteries are not considered in cbPWV calculus). As can be seen in the figure, crPWV data obtained in this work and those obtained for cbPWV by McEniery et al. showed similar profiles, although the levels of the former were higher. The lower cbPWV levels (compared to those of crPWV) could contribute to explain the differences in the age at which a PWV Ratio = 1 was observed. In other words, the more proximal the arteries or arterial territories measured, the earlier (in terms of years) the PWV ratio would reach a value = 1. On the other hand, it was difficult to carry out a similar analysis considering Niiranen et al. data because although a particular value is shown in the graph (e.g., 35 y), data published by the authors were the mean of a decade (e.g., 40–49 y, 50–59 y, 60–69 y) [53] (Figure 8).

Both cfPWV and crPWVcurves showed the expected behavior. About this, cfPWV evidenced the expected curvilinear relationship between age and aortic stiffness (Figure 8), in which age-related stiffness changes were less marked in middle-aged adults and became gradually greater after ~60 y. The steep rise in aortic PWV after the 5–6th decade mirrored the age-related widening in brachial pulse pressure, previously described in nearly all populations worldwide. In contrast to the factors described for aortic stiffness, it is recognized that peripheral (muscular) arteries are less affected by the aging process. About this, age-related increases in crPWV and cbPWV were more gradual, and the range of variation (p50) for crPWV or cbPWV was smaller than for cfPWV (Figure 8).

**Figure 9:**
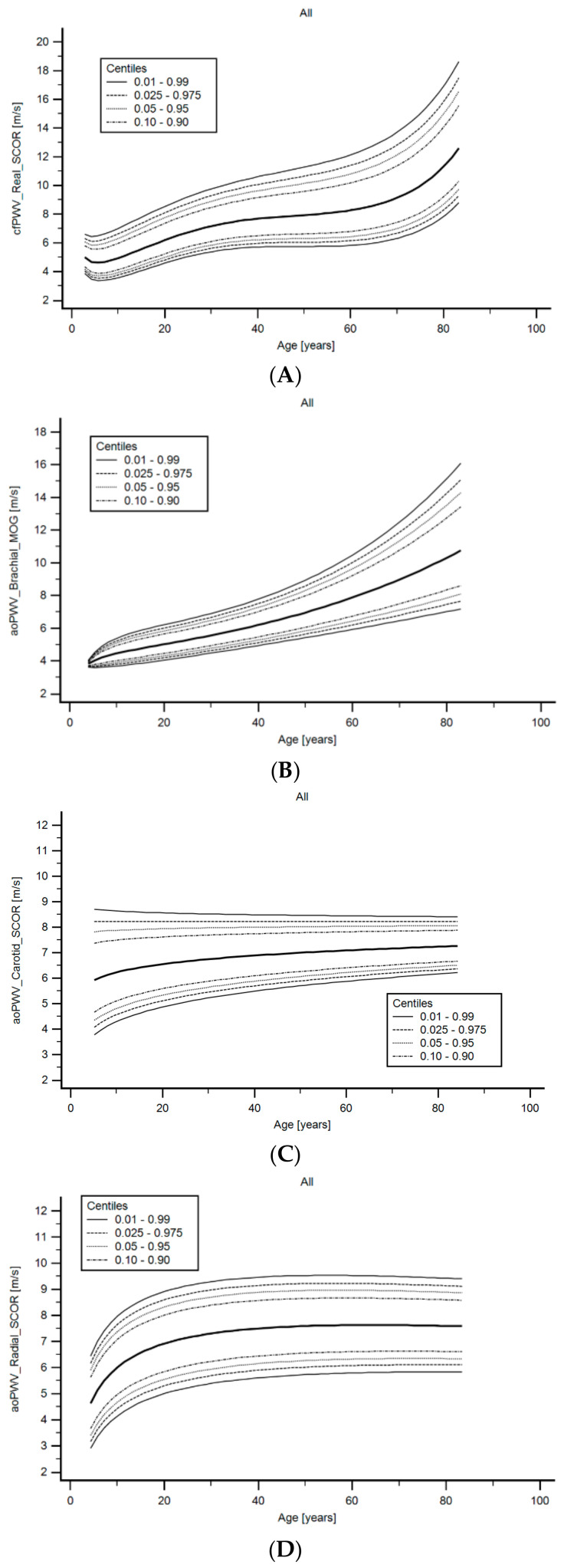
cfPWV_Real_SCOR (**A**), aoPWV_Brachial_MOG (**B**), aoPWV_Carotid_SCOR (**C**) and aoPWV_Radial_SCOR (**D**) age-related profiles for subjects included in the RI group.

Finally, the different approaches used to assess aortic stiffness did not show similar age-related profiles (Figure 9). While cfPWV_Real_SCOR and aoPWV_Brachial_MOG showed the expected profile (described above), aoPWV_Carotid_SCOR and aoPWV_Radial_SCOR, showed a different behavior, with a clear rise in the first years (also seen for cfPWV_Real_SCOR), followed by a low rate increase (almost flat) (Figure 9). Then, aoPWV_Carotid_SCOR and aoPWV_Radial_SCOR, would not show the (expected) age-related stiffness profile.

### 4.2. Strengths and Limitations

Our results should be interpreted within the context of this work’s strengths and limitations. As the study is a cross-sectional one, it provides no data on longitudinal age-related changes in cfPWV, crPWV or aoPWV (and hence in CAVI and CAVIo). Since no outcome data were considered, cut-off points (e.g., p75th, p90th, p95th) were not defined based on CV risk but on value distribution in the RIs group. Whether or not the reference values should be used as cutoff values for diagnosis and treatment is not known. In this work, the concept of arterial stiffness is mainly presented as a “static process”, rather than the composite of (i) fixed (e.g., due to fibrosis or arterial wall remodeling) and (ii) variable (e.g., due to vascular smooth muscle (VSM) activation-related arterial stiffness (viscoelasticity) modulation). Two issues should be considered in this regard. First, the increases in arterial stiffness with increasing age do not necessarily reflect “concrete-like” changes, but rather a variable and less known increase in stiffness associated with increases in VSM tone (e.g., increased constrictor tone) [54,55]. This component would be of particular importance in the treatment of both hypertension and/or heart failure, and thus represents a potentially central goal in drug therapy [54]. Second, for any age, arterial stiffness can be acutely and temporarily modified by variations in the VSM tone [55,56,57,58]. Systematization of recording conditions is necessary for the evaluation of arterial stiffness considering the modulating role of the VSM tone. In this work, to systematize the records and as a way to minimize the impact of the referred source of variability, arterial stiffness levels were assessed and determined at rest, under stable hemodynamic state conditions. A major strength of the study is that arterial stiffness parameters were obtained in a large population sample (including children, adolescents and adults), and defines the trends in mean value and percentiles for almost the whole range of life expectancy. This would help to identify the physiological behavior (levels and rates) of arterial stiffness and center-to-periphery gradient throughout life, providing important information for clinical diagnosis.

## 5. Conclusions

Methods and parameters used to assess aortic stiffness (cfPWV_Real, aoPWV_Radial, aoPWV_Carotid and aoPWV_Brachial) and their CAVI and CAVIo, showed different association levels, which in no case were very strong (r ≥ 0.80). Data from different approaches were not equivalent but showed systematic and proportional errors.

The need to define sex-specific RIs relied on the approach used to assess the stiffness and/or on the age considered. In this work, population-based reference values for PWV, stiffness gradient, CAVI and CAVIo indexes were defined; a key step when considering the introduction of the indexes as a tool for the detection of subclinical target organ damage.

## Figures and Tables

**Table 1:** PWV-, CAVI- and CAVIo-related parameters.

PWV-Related Parameter	CAVI-Related Parameter	CAVIo-Related Parameter
cfPWV_Real_SCOR	CAVI_cfPWV_Real_SCOR	CAVIo_cfPWV_Real_SCOR
crPWV_SCOR	CAVI_crPWV_SCOR	CAVIo_crPWV_SCOR
aoPWV_Radial_SCOR	CAVI_aoPWV_Radial_SCOR	CAVIo_aoPWV_Radial_SCOR
aoPWV_Carotid_SCOR	CAVI_aoPWV_Carotid_SCOR	CAVIo_aoPWV_Carotid_SCOR
aoPWV_Brachial_MOG	CAVI_aoPWV_Brachial_MOG	CAVIo_aoPWV_Brachial_MOG
PWV Ratio	CAVI_PWV_Ratio	CAVIo_PWV_Ratio

**Table 2:** Subjects demographic, anthropometric and clinical characteristics.

	All Subjects (*n* = 3619)								Reference Intervals Group (*n* = 1289)							
	MV	SD	Min.	p25th	p50th	p75th	Max.	Range	MV	SD	Min.	p25th	p50th	p75th	Max.	Range
Age (years)	33.9	24.2	2.8	11.5	23.7	56.4	89.0	86.2	20.1	16.9	2.8	6.3	17.6	21.8	84.2	81.4
BW (kg)	61.1	25.3	12.3	45.6	63.2	78.1	150.6	138.3	47.9	22.8	12.3	22.6	52.8	65.2	105.0	92.7
BH (m)	1.55	0.23	0.90	1.46	1.62	1.71	1.97	1.07	1.47	0.26	0.90	1.17	1.58	1.69	1.94	1.04
BMI (kg/m^2^)	24.06	6.02	11.53	19.7	23.63	27.84	71.34	59.81	20.36	4.22	11.53	16.59	20	23.56	29.95	18.42
z-BMI (SD) *	0.94	1.45	−4.63	−0.05	0.74	1.77	8.03	12.66	0.34	0.92	−4.63	−0.27	0.41	1	1.98	6.61
TC (mg/dL)	200	44	94	170	196	227	379	285	195	26	99	179	198	214	238	139
HDL (mg/dL)	51	15	17	41	49	60	122	105	58	12	41	49	55	64	100	59
LDL (mg/dL)	123	40	28	95	119	146	323	295	118	26	31	101	121	134	180	149
TG (mg/dL)	133	86	24	80	111	158	783	759	93	39	24	65	86	113	272	248
Glic. (mg/dL)	94	19	40	85	92	100	307	267	88	9	40	83	88	93	121	81
bSBP (mmHg)	119	17	64	107	118	129	235	171	112	13	80	101	112	121	171	91
bDBP (mmHg)	69	10	41	61	68	76	129	88	65	8	47	59	63	70	97	51
TC ≥240 mg/dL (%)	7.20								0.00							
HDL <40 mg/dL (%)	8.90								0.00							
Glic. ≥126 (%) mg/dL	0.90								0.00							
Current Smoker (%)	11.40								0.00							
Hypertension (%)	26.40								0.00							
Diabetes (%)	5.70								0.00							
History of CVD (%)	8.80								0.00							
Obesity (%)	22.60								0.00							
Family CVD (%)	13.50								7.60							
Sedentarism (%)	45.60								32.30							
Anti-hypertensive (%)	21.70								0.00							
Anti-hyperlip. (%)	13.50								0.00							
Anti-diabetic (%)	4.10								0.00							
Atheroma plaques (%)																
0	64.70								88.20							
1	6.20								2.70							
2	8.10								4.20							
3	6.40								1.90							
4	6.50								1.80							
5	3.20								0.60							
6	3.00								0.30							
7	1.20								0.10							
8	0.70								0.10							

MV: mean value. SD: standard deviation. Min. and Max.: minimum and maximum values. p25th, p50th (median) and p75th: 25, 50 and 75 percentiles. BMI: body mass index. bSBP, bDBP: brachial systolic and diastolic blood pressure. CVD: cardiovascular disease. TC: total cholesterol. TG: Triglycerides. Glic: Glycemia. Hyperlip: hyperlipidemic Atheroma plaques refers to plaques in common, internal and external carotid arteries; common femoral arteries (both hemibodies; 8 segments); presented as% of subjects with 0, 1, 2, ...., 8 segments affected. * only for <18 y. BW: body weight. BH: Body height.

**Table 3:** Arterial stiffness parameters.

	All Subjects (*n* = 3619)								Reference Intervals Group (*n* = 1289)							
	MV	SD	Min.	p25th	p50th	p75th	Max.	Range	MV	SD	Min.	p25th	p50th	p75th	Max.	Range
**cfPWV_Real_SCOR**																
bSBP (mmHg)	120	17	63	110	120	130	211	148	114	14	79	103	113	122	167	88
bDBP (mmHg)	69	11	40	60	69	76	127	87	64	9	40	58	63	70	100	60
HR (b.p.m.)	73	15	35	62	71	82	149	114	75	15	40	64	74	85	134	94
cfPWV_Real_SCOR (m/s)	7.39	2.46	2.7	5.57	7	8.72	19.75	17.06	6.23	1.63	2.7	5.04	5.98	7.13	14.59	11.89
CAVI_cfPWV_Real_SCOR	10.06	6.6	1.61	5.73	8.3	12.32	65.22	63.62	7.34	3.56	1.61	4.97	6.57	8.84	33.43	31.82
CAVIo_cfPWV_Real_SCOR	13.89	9.09	2.62	8.2	11.45	16.47	97.39	94.77	10.31	4.68	2.7	7.13	9.36	12.24	46.84	44.14
**crPWV_SCOR**																
bSBP (mmHg)	120	15	71	110	119	129	195	124	115	13	71	106	114	124	162	91
bDBP (mmHg)	68	10	40	60	68	76	102	62	65	9	43	58	64	70	100	57
HR (b.p.m.)	71	13	35	61	69	79	140	105	72	14	40	62	70	81	131	91
crPWV_SCOR (m/s)	9.3	1.97	4.4	7.8	9.2	10.7	15.7	11.3	8.76	1.76	4.4	7.5	8.5	9.9	14.9	10.5
CAVI_crPWV_SCOR	15.47	5.6	3.11	11.32	15.03	19.04	47.5	44.39	14.38	5.18	4.46	10.87	13.35	17.27	47.5	43.04
CAVIo_crPWV_SCOR	21.11	7.14	3.8	15.9	20.72	25.59	63.07	59.28	19.86	6.72	6.79	15.18	18.73	23.49	63.07	56.28
**PWV Ratio (cfPWV_Real_SCOR/crPWV_SCOR)**																
PWV_Ratio	0.78	0.19	0.35	0.65	0.74	0.87	1.86	1.51	0.72	0.15	0.35	0.62	0.71	0.8	1.49	1.14
CAVI_PWV_Ratio	0.64	0.35	0.12	0.42	0.56	0.76	3.67	3.55	0.54	0.23	0.12	0.38	0.5	0.64	2.3	2.18
CAVIo_PWV_Ratio	0.65	0.34	0.13	0.43	0.57	0.76	3.87	3.74	0.55	0.23	0.13	0.39	0.52	0.65	2.36	2.23
**aoPWV_Radial_SCOR**																
bSBP (mmHg)	121	16	77	109	120	130	235	158	114	14	78	105	114	124	160	82
bDBP (mmHg)	69	11	37	61	69	76	130	93	65	9	42	59	64	71	95	53
HR (b.p.m.)	73	14	35	63	71	82	151	116	76	15	38	65	74	85	151	113
aoPWV_Radial_SCOR (m/s)	7.24	1.25	4	6.5	7.2	8	14.3	10.3	6.73	1.15	4	6	6.8	7.4	11.2	7.2
CAVI_aoPWV_Radial_SCOR	9.21	2.92	3.16	7.31	8.8	10.58	33.55	30.39	8.41	2.62	3.37	6.72	8.2	9.68	21.7	18.33
CAVIo_aoPWV_Radial_SCOR	12.82	4.07	4.51	10.12	12.28	14.76	46.81	42.3	11.9	3.71	4.51	9.56	11.44	13.77	28.85	24.34
**aoPWV_Carotid_SCOR**																
bSBP (mmHg)	121	18	78	109	120	131	239	161	114	15	78	104	114	124	217	139
bDBP (mmHg)	69	11	38	60	68	76	127	89	64	9	38	58	63	70	100	62
HR (b.p.m.)	72	14	32	61	70	80	145	113	75	15	40	64	74	84	145	105
aoPWV_Carotid_SCOR (m/s)	7.01	1.02	4	6.4	7	7.6	13.2	9.2	6.68	0.97	4	6.2	6.6	7.2	13.2	9.2
CAVI_aoPWV_Carotid_SCOR	8.51	2.44	2.94	6.95	8.11	9.58	29.7	26.76	8.08	2.42	2.94	6.68	7.73	8.98	28.59	25.65
CAVIo_aoPWV_Carotid_SCOR	11.85	3.46	3.99	9.66	11.25	13.41	40.02	36.03	11.47	3.54	3.99	9.37	10.84	12.79	39.94	35.95
**aoPWV_Brachial_MOG**																
bSBP (mmHg)	117	14	81	107	115	125	199	118	112	12	81	104	111	120	158	77
bDBP (mmHg)	68	11	36	60	67	75	131	95	65	9	39	58	64	70	106	67
HR (b.p.m.)	76	15	33	64	73	86	135	102	79	16	41	67	77	89	135	94
aoPWV_Brachial_MOG (m/s)	5.74	2.07	3.58	4.33	4.88	6.36	15.25	11.68	4.87	1.18	3.58	4.15	4.55	5.08	12.93	9.36
CAVI_aoPWV_Brachial_MOG	6.26	4.55	2.84	3.58	4.21	6.7	31.45	28.62	4.55	2.46	2.84	3.4	3.83	4.55	28.09	25.25
CAVIo_aoPWV_Brachial_MOG	8.64	5.84	3.86	5.21	6.25	9.15	46.01	42.15	6.51	3.19	3.9	4.92	5.57	6.74	38.9	35

Abbreviations: similar to Table 1. HR: heart rate. Other abbreviations: see the text.

**Table 4:** Concordance correlation coefficients between PWV parameters (Reference Interval Group).

**cfPWV (Variable Y) vs. aoPWV_Radial_SCOR, aoPWV_Carotid_SCOR or aoPWV_Brachial_MOG**			
Variable X	aoPWV_Radial_SCOR	aoPWV_Carotid_SCOR	aoPWV_Brachial_MOG
CCC	0.448	0.271	0.614
95% C.I. CCC	0.3963 to 0.4967	0.2073 to 0.3319	0.5727 to 0.6527
Pearson ρ (precision)	0.498	0.3145	0.7336
*p*-value (Pearson)	<1.0 × 10^−14^	<1.0 × 10^−14^	<1.0 × 10^−14^
**aoPWV_Radial_SCOR vs. aoPWV_Carotid_SCOR vs. aoPWV_Brachial_MOG**			
Variable Y	aoPWV_Radial_SCOR	aoPWV_Radial_SCOR	aoPWV_Carotid_SCOR
Variable X	aoPWV_Carotid_SCOR	aoPWV_Brachial_MOG	aoPWV_Brachial_MOG
CCC	0.617	0.235	0.109
95% C.I. CCC	0.5661 to 0.6632	0.1851 to 0.2838	0.04981 to 0.1668
Pearson ρ (precision)	0.6425	0.4205	0.1882
*p*-value (Pearson)	<1.0 × 10^−14^	<1.0 × 10^−14^	0.000257
**CAVI_cfPWV (Variable Y) vs. CAVI_aoPWV: Radial_SCOR, Carotid_SCOR or Brachial_MOG**			
Variable Y	CAVI_cfPWV_Real_SCOR	CAVI_cfPWV_Real_SCOR	CAVI_cfPWV_Real_SCOR
Variable X	CAVI_aoPWV_Radial_SCOR	CAVI_aoPWV_Carotid_SCOR	CAVI_aoPWV_Brachial_MOG
CCC	0.2779	0.09839	0.553
95% C.I. CCC	0.2181 to 0.3355	0.02888 to 0.1670	0.5073 to 0.5956
Pearson ρ (precision)	0.3075	0.1115	0.6704
*p*-value (Pearson)	<1.0 × 10^−14^	0.0057	<1.0 × 10^−14^
**CAVI_aoPWV_Radial_SCOR vs. CAVI_aoPWV_Carotid_SCOR vs. CAVI_aoPWV_Brachial_MOG**			
Variable Y	CAVI_aoPWV_Radial_SCOR	CAVI_aoPWV_Radial_SCOR	CAVI_aoPWV_Carotid_SCOR
Variable X	CAVIo_aoPWV_Carotid_SCOR	CAVIo_aoPWV_Brachial_MOG	CAVIo_aoPWV_Brachial_MOG
CCC	0.5133	0.1439	0.03279
95% C.I. CCC	0.4538 to 0.5682	0.09252 to 0.1944	−0.03191 to 0.09722
Pearson ρ (precision)	0.5389	0.2545	0.05159
*p*-value (Pearson)	<1.0 × 10^−14^	2.36 × 10^−8^	0.3203
**CAVIo_cfPWV (variable Y) vs. CAVIo_aoPWV: Radial_SCOR, Carotid_SCOR or Brachial_MOG**			
Variable X	CAVIo_aoPWV_Radial_SCOR	CAVIo_aoPWV_Carotid_SCOR	CAVIo_aoPWV_Brachial_MOG
CCC	0.2294	0.08023	0.5078
95% C.I. CCC	0.1671 to 0.2898	0.007051 to 0.1526	0.4608 to 0.5518
Pearson ρ (precision)	0.25	0.08661	0.6402
*p*-value (Pearson)	2.02E−12	0.0319	1.00 × 10^−14^
**CAVIo_aoPWV_Radial_SCOR vs. CAVIo_aoPWV_Carotid_SCOR vs. CAVIo_aoPWV_Brachial_MOG**			
Variable Y	CAVIo_aoPWV_Radial_SCOR	CAVIo_aoPWV_Radial_SCOR	CAVIo_aoPWV_Carotid_SCOR
Variable X	CAVIo_aoPWV_Carotid_SCOR	CAVIo_aoPWV_Brachial_MOG	CAVIo_aoPWV_Brachial_MOG
CCC	0.5623	0.08684	0.008803
95% C.I. CCC	0.5064 to 0.6134	0.03752 to 0.1357	−0.05276 to 0.07029
Pearson ρ (precision)	0.5864	0.1604	0.01454
*p*-value (Pearson)	<1.0 × 10^−14^	4.97 × 10^−4^	7.80 × 10^−1^

CCC: concordance correlation coefficient. C.I.: confidence interval.

**Table 5:** Bland–Altman test between arterial stiffness parameters (Reference Interval Group).

**Part 1**									
**(1) cfPWV (Method A) vs. aoPWV: Radial_SCOR, Carotid_SCOR or Brachial_MOG**									
Method B			aoPWV_Radial_SCOR			aoPWV_Carotid_SCOR			aoPWV_Brachial_MOG
**Differences (Method A-Method B)**									
Mean error (m/s)			−0.41			−0.14			0.81
Mean error, 95% CI (m/s)			−0.5123 to −0.3052			−0.2624 to −0.009219			0.7308 to 0.8833
Mean error, P (H_0_: Mean = 0)			2.99 × 10^−14^			3.55 × 10^−2^			<1.0 × 10^−14^
Mean error, Lower limit (m/s)			−3.2767			−3.2661			−1.1438
Mean error, 95% CI (m/s)			−3.4538 to −3.0996			−3.4826 to −3.0497			−1.2742 to −1.0134
Mean error, Upper limit (m/s)			2.4592			2.9945			2.7578
Mean error, 95% CI (m/s)			2.2821 to 2.6363			2.7781 to 3.2110			2.6274 to 2.8882
Mean error, Regression Eq.			y = −3.5184 + 0.4766x			y =−5.3391 + 0.7875x			y = −0.1298 + 0.1732x
**Parameter**									
Intercept, Coeff. (m/s)			−3.5184			−5.3391			−0.1298
Intercept, Coeff. 95% CI (m/s)			−4.0397 to −2.9971			−6.0251 to −4.6530			−0.4615 to 0.2019
Intercept, Coeff. *p*-value			<1.0 × 10^−14^			<1.0 × 10^−14^			0.4425
**Slope**									
Slope, Coeff. (m/s/y)			0.4766			0.7875			0.1732
Slope, Coeff. 95% CI (m/s/y)			0.3980 to 0.5551			0.6850 to 0.8900			0.1135 to 0.2330
Slope, Coeff. *p*-value			<1.0 × 10^−14^			<1.0 × 10^−14^			0.000
**(2) aoPWV_Radial_SCOR vs. aoPWV_Carotid_SCOR vs. aoPWV_Brachial_MOG**									
Method A			aoPWV_Radial_SCOR			aoPWV_Radial_SCOR			aoPWV_Carotid_SCOR
Method B			aoPWV_Carotid_SCOR			aoPWV_Brachial_MOG			aoPWV_Brachial_MOG
**Differences (Method A-Method B)**									
Mean error (m/s)			0.28			1.49			1.38
Mean error, 95% CI (m/s)			0.2122 to 0.3457			1.3754 to 1.6096			1.2300 to 1.5345
Mean error, P (H_0_: Mean = 0)			<1.0 × 10^−14^			<1.0 × 10^−14^			<1.0 × 10^−14^
Mean error, Lower limit (m/s)			−1.3327			−1.0341			−1.5492
Mean error, 95% CI (m/s)			−1.4469 to −1.2185			−1.2344 to −0.8339			−1.8097 to −1.2887
Mean error, Upper limit (m/s)			1.8907			4.0192			4.3137
Mean error, 95% CI (m/s)			1.7765 to 2.0049			3.8189 to 4.2194			4.0532 to 4.5742
Mean error, Regression Eq.			y = 0.2207 + 0.008533x			y = 2.2722−0.1320x			y = 3.3683−0.3309x
**Intercept**									
Intercept, Coeff. (m/s)			0.2207			2.2722			3.3683
Intercept, Coeff. 95% CI (m/s)			−0.3024 to 0.7437			1.5775 to 2.9670			2.3609 to 4.3756
Intercept, Coeff. *p*-value			0.4077			3.23 × 10^−10^			1.65 × 10^−10^
**Slope**									
Slope, Coeff. (m/s/y)			0.008533			−0.132			−0.3309
Slope, Coeff. 95% CI (m/s/y)			−0.06737 to 0.08444			−0.2479 to −0.01605			−0.4969 to −0.1649
Slope, Coeff. *p*-value			0.8253			0.0258			0.000
**Part 2**									
**(3) CAVI_cfPWV_Real_SCOR (Method A) vs. CAVI_aoPWV: Radial_SCOR, Carotid_SCOR or Brachial_MOG**									
Method B		CAVI_aoPWV_Radial_SCOR			CAVI_aoPWV_Carotid_SCOR			CAVI_aoPWV_Brachial_MOG	
**Differences (Method A-Method B)**									
Mean error		−0.89			−0.12			1.81	
Mean error, 95% CI		−1.1609 to −0.6148			−0.4612 to 0.2183			1.6231 to 1.9921	
Mean error, P (H_0_: Mean = 0)		3.00 × 10^−10^			4.83 × 10^−1^			<1.0 × 10^−14^	
Mean error, Lower limit		−8.4481			−8.5235			−2.9128	
Mean error, 95% CI		−8.9150 to −7.9813			−9.1045 to −7.9426			−3.2283 to −2.5973	
Mean error, Upper limit		6.6724			8.2806			6.5281	
Mean error, 95% CI		6.2055 to 7.1392			7.6997 to 8.8615			6.2126 to 6.8436	
Mean error, Regression Eq.		y = −5.1891 + 0.5399x			y = −6.9488 + 0.8540x			y = 0.5711 + 0.2188x	
**Intercept**									
Intercept, Coeff.		−5.1891			−6.9488			0.5711	
Intercept, Coeff. 95% CI		−6.0114 to −4.3667			−8.0063 to −5.8912			0.1496 to 0.9925	
Intercept, Coeff. *p*-value		<1.0 × 10^−14^			<1.0 × 10^−14^			0.008	
**Slope**									
Slope, Coeff.		0.5399			0.854			0.2188	
Slope, Coeff. 95% CI		0.4418 to 0.6381			0.7272 to 0.9809			0.1513 to 0.2862	
Slope, Coeff. *p*-value		<1.0 × 10^−14^			<1.0 × 10^−14^			<0.0001	
**(4) CAVI_aoPWV_Radial_SCOR vs. CAVI_aoPWV_Carotid_SCOR vs. CAVI_aoPWV_Brachial_MOG**									
Method A		CAVI_aoPWV_Radial_SCOR			CAVI_aoPWV_Radial_SCOR			CAVI_aoPWV_Carotid_SCOR	
Method B		CAVI_aoPWV_Carotid_SCOR			CAVI_aoPWV_Brachial_MOG			CAVI_aoPWV_Brachial_MOG	
**Differences (Method A-Method B)**									
Mean error		0.76			3.34			3.02	
Mean error, 95% CI		0.5752 to 0.9524			3.0420 to 3.6416			2.6246 to 3.4233	
Mean error, P (H_0_: Mean = 0)		<1.0 × 10^−14^			<1.0 × 10^−14^			<1.0 × 10^−14^	
Mean error, Lower limit		−3.7885			−3.1272			−4.6633	
Mean error, 95% CI		−4.1111 to −3.4660			−3.6399 to −2.6145			−5.3464 to −3.9803	
Mean error, Upper limit		5.3161			9.8107			10.7112	
Mean error, 95% CI		4.9936 to 5.6386			9.2980 to 10.3235			10.0282 to 11.3943	
Mean error, Regression Eq.		y = 0.8012 − 0.004401x			y = 3.9494 − 0.09117x			y = 4.3010 − 0.1895x	
**Intercept**									
Intercept, Coeff.		0.8012			3.9494			4.301	
Intercept, Coeff. 95% CI		0.02191 to 1.5804			2.9684 to 4.9304			2.9409 to 5.6611	
Intercept, Coeff. *p*-value		0.0439			1.87 × 10^−14^			1.36 × 10^−9^	
**Slope**									
Slope, Coeff.		−0.004401			−0.09117			−0.1895	
Slope, Coeff. 95% CI		−0.09345 to 0.08465			−0.2313 to 0.04899			−0.3825 to 0.003503	
Slope, Coeff. *p*-value		0.9227			0.2018			0.054	
**Part 3**									
**(5) CAVIo_cfPWV_Real_SCOR (Method A) vs. CAVIo_aoPWV: Radial_SCOR, Carotid_SCOR orBrachial_MOG**									
Method B	CAVIo_aoPWV_Radial_SCOR			CAVIo_aoPWV_Carotid_SCOR			CAVIo_aoPWV_Brachial_MOG		
**Differences (Method A-Method B)**									
Mean error	−1.29			−0.28			2.63		
Mean error, 95% CI	−1.6699 to −0.9076			−0.7459 to 0.1913			2.3706 to 2.8798		
Mean error, P (H_0_: Mean = 0)	6.03 × 10^−11^			0.2456			<1.0 × 10^−14^		
Mean error, Lower limit	−11.8424			−11.8658			−3.8891		
Mean error, 95% CI	−12.4942 to −11.1907			−12.6671 to −11.0646			−4.3245 to −3.4538		
Mean error, Upper limit	9.2649			11.3112			9.1395		
Mean error, 95% CI	8.6132 to 9.9166			10.5100 to 12.1125			8.7041 to 9.5748		
Mean error, Regression Eq.	y = −6.5225 + 0.4651x			y = −8.0619 + 0.6887x			y = 0.5729 + 0.2545x		
**Intercept**									
Intercept, Coeff.	−6.5225			−8.0619			0.5729		
Intercept, Coeff. 95% CI	−7.7702 to −5.2748			−9.6582 to −6.4655			−0.04940 to 1.1952		
Intercept, Coeff. *p*-value	<1.0 × 10^−14^			<1.0 × 10^−14^			0.0711		
**Slope**									
Slope, Coeff.	0.4651			0.6887			0.2545		
Slope, Coeff. 95% CI	0.3591 to 0.5712			0.5528 to 0.8246			0.1836 to 0.3255		
Slope, Coeff. *p*-value	<1.0 × 10^−14^			<1.0 × 10^−14^			4.61 × 10^−12^		
**(6) CAVIo_aoPWV_Radial_SCOR vs. CAVIo_aoPWV_Carotid_SCOR vs. CAVIo_aoPWV_Brachial_MOG**									
Method A	CAVIo_aoPWV_Radial_SCOR			CAVIo_aoPWV_Radial_SCOR			CAVIo_aoPWV_Carotid_SCOR		
Method B	CAVIo_aoPWV_Carotid_SCOR			CAVIo_aoPWV_Brachial_MOG			CAVIo_aoPWV_Brachial_MOG		
**Differences (Method A-Method B)**									
Mean error	1.02			4.82			4.45		
Mean error, 95% CI	0.7615 to 1.2788			4.3819 to 5.2562			3.8948 to 5.0112		
Mean error, P (H_0_: Mean = 0)	4.22 × 10^−14^			<1.0 × 10^−14^			<1.0 × 10^−14^		
Mean error, Lower limit	−5.2235			−4.6138			−6.2933		
Mean error, 95% CI	−5.6658 to −4.7812			−5.3615 to −3.8662			−7.2482 to −5.3384		
Mean error, Upper limit	7.2638			14.2519			15.1993		
Mean error, 95% CI	6.8215 to 7.7061			13.5042 to 14.9995			14.2444 to 16.1542		
Mean error, Regression Eq.	y = 1.4747 − 0.03782x			y = 3.9543 + 0.09114x			y = 4.3849 + 0.007097x		
**Intercept**									
Intercept, Coeff.	1.4747			3.9543			4.3849		
Intercept, Coeff. 95% CI	0.4438 to 2.5056			2.4230 to 5.4856			2.3751 to 6.3948		
Intercept, Coeff. *p*-value	0.0051			0.000000562			0.0000228		
**Slope**									
Slope, Coeff.	−0.03782			0.09114			0.007097		
Slope, Coeff. 95% CI	−0.1209 to 0.04522			−0.06354 to 0.2458			−0.1941 to 0.2083		
Slope, Coeff. *p*-value	0.3714			0.2475			0.9447		

Eq.: Equation. C.I.: confidence interval. SE: standard error. m: meter. s: second. y: year. Coeff.: Coefficient.

**Table 6:** Association between stiffness parameter and plaque presence or sex (adjusted by age and/or sex).

				aoPWV		
		cfPWV_Real_SCOR (m/s)	crPWV_SCOR (m/s)	Radial_SCOR (m/s)	Carotid_SCOR (m/s)	Brachial_MOG (m/s)
Atherosclerotic plaques (Yes: 1; No:0) (*)	r *	0.107	−0.109	−0.054	−0.034	0.090
	*p* (2-tailed)	0.072	0.066	0.362	0.563	0.130
	Boot, Bias	−0.003	0.001	−0.002	−0.002	0.003
	Boot, SE	0.085	0.057	0.057	0.035	0.097
	Boot 95%CI LL	−0.069	−0.216	−0.166	−0.102	−0.100
	Boot 95%CI UL	0.269	−0.002	0.056	0.037	0.283
Atherosclerotic plaques (Yes: 1; No:0] (**)	r **	0.104	−0.108	−0.052	−0.036	0.086
	*p* (2-tailed)	0.080	0.071	0.384	0.552	0.149
	Boot, Bias	−0.007	−0.003	0.002	−0.001	0.004
	Boot, SE	0.090	0.055	0.062	0.036	0.104
	Boot 95%CI LL	−0.082	−0.222	−0.169	−0.104	−0.110
	Boot 95%CI UL	0.272	−0.006	0.070	0.037	0.294
Sex (Female: 1; Male:0) (*)	r *	−0.123	0.079	0.091	−0.038	−0.224
	*p* (2-tailed)	0.039	0.184	0.127	0.526	0.000
	Boot, Bias	0.002	0.003	0.002	−0.001	−0.005
	Boot, SE	0.056	0.059	0.055	0.060	0.060
	Boot 95%CI, LL	−0.229	−0.038	−0.016	−0.153	−0.351
	Boot 95%CI UL	−0.009	0.192	0.194	0.076	−0.115

(*) adjusted by age (years). (**) adjusted by age (years) and sex (Female: 1; Male: 0). Boot: bootstrap. SE: standard error. CI: confidence interval. LL: lower limit. UL: Upper limit.

**Table 7:** (Part 1). Interaction between age and sex as a determinant of cfPWV_Real_SCOR (m/s). (Part 2). Interaction between age and sex as a determinant of crPWV_SCOR (m/s). (Part 3). Interaction between age and sex as a determinant of aoPWV values.

**Part 1**															
**Model Summary (y: cfPWV_Real_SCOR; x: Sex; w: Age): R = 0.803; R^2^ = 0.6534; *p* = <0.0001**															
	Coeff			SE				p			95% CI LL			95% CI UL	
Intercept	4.4829			0.0617				<0.0001			4.3618			4.604	
Sex (Female: 1; Male: 0)	0.0641			0.0882				0.4671			−0.1088			0.2371	
Age (years)	0.0784			0.0021				<0.0001			0.0743			0.0825	
Sex * Age	−0.0087			0.003				0.0041			−0.0146			−0.0028	
**Moderator value(s) defining Johnson-Neyman significance region(s)**															
Value (years)	% below			% above											
19.6737	60.4344			39.5656											
**Conditional effect of focal predictor at values of the moderator**															
Age (years)	Effect			SE				p			95% CI LL			95% CI UL	
2.80	0.0398			0.0816				0.626			−0.1203			0.1998	
6.87	0.0044			0.0727				0.952			−0.1384			0.1471	
10.94	−0.031			0.065				0.633			−0.1586			0.0966	
15.01	−0.0665			0.0589				0.260			−0.1821			0.0491	
19.08	−0.1019			0.0549				0.064			−0.2096			0.0059	
19.67	−0.107			0.0546				0.050			−0.2141			0	
23.15	−0.1373			0.0535				0.011			−0.2423			−0.0323	
27.22	−0.1727			0.0549				0.002			−0.2804			−0.0649	
31.29	−0.2081			0.0589				0.000			−0.3237			−0.0925	
35.36	−0.2435			0.065				0.000			−0.3711			−0.1159	
39.43	−0.2789			0.0728				0.000			−0.4216			−0.1362	
43.50	−0.3143			0.0816				0.000			−0.4744			−0.1542	
47.57	−0.3497			0.0913				0.000			−0.5288			−0.1707	
51.64	−0.3851			0.1015				0.000			−0.5842			−0.186	
55.71	−0.4205			0.1121				0.000			−0.6405			−0.2005	
59.78	−0.456			0.1231				0.000			−0.6975			−0.2144	
63.85	−0.4914			0.1343				0.000			−0.7548			−0.2279	
67.92	−0.5268			0.1457				0.000			−0.8126			−0.241	
71.99	−0.5622			0.1572				0.000			−0.8706			−0.2538	
76.06	−0.5976			0.1688				0.000			−0.9288			−0.2664	
80.13	−0.633			0.1806				0.001			−0.9872			−0.2788	
84.20	−0.6684			0.1924				0.001			−1.0458			−0.291	
**Part 2**															
**Model Summary (y: crPWV_SCOR; x: Sex; w: Age): R = 0.5664; R^2^ = 0.3208; *p* = <0.0001**															
		Coeff			SE				P	95% CI LL			95% CI UL		
Intercept		7.3354			0.1244				0.000	7.0913			7.5796		
Sex (Female: 1; Male: 0)		0.1806			0.1831				0.324	−0.1789			0.5401		
Age (years)		0.0615			0.0042				0.000	0.0532			0.0697		
Sex * Age		−0.0139			0.006				0.022	−0.0257			−0.002		
**Moderator value(s) defining Johnson-Neyman significance region(s):**															
Value		% below			% above										
28.9325 (years)		74.3017			25.6983										
**Conditional effect of focal predictor at values of the moderator:**															
Age (years)		Effect			SE				P	95% CI LL			95% CI UL		
5.20		0.1085			0.159				0.496	−0.2038			0.4207		
9.15		0.0536			0.1427				0.707	−0.2265			0.3337		
13.10		−0.0012			0.1286				0.993	−0.2537			0.2513		
17.05		−0.056			0.1178				0.634	−0.2872			0.1752		
21.00		−0.1109			0.111				0.319	−0.3289			0.1072		
24.95		−0.1657			0.1092				0.130	−0.3801			0.0487		
28.90		−0.2205			0.1125				0.050	−0.4414			0.0004		
28.93		−0.221			0.1125				0.050	−0.4419			0		
32.85		−0.2753			0.1205				0.023	−0.5119			−0.0388		
36.80		−0.3302			0.1324				0.013	−0.59			−0.0703		
40.75		−0.385			0.1471				0.009	−0.6739			−0.0961		
44.70		−0.4398			0.1641				0.008	−0.7619			−0.1177		
48.65		−0.4946			0.1825				0.007	−0.853			−0.1363		
52.60		−0.5495			0.2021				0.007	−0.9463			−0.1526		
56.55		−0.6043			0.2225				0.007	−1.0412			−0.1674		
60.50		−0.6591			0.2436				0.007	−1.1373			−0.1809		
64.45		−0.714			0.2651				0.007	−1.2344			−0.1935		
68.40		−0.7688			0.2869				0.008	−1.3322			−0.2054		
72.35		−0.8236			0.3091				0.008	−1.4305			−0.2167		
76.30		−0.8784			0.3315				0.008	−1.5293			−0.2276		
80.25		−0.9333			0.3541				0.009	−1.6284			−0.2381		
84.20		−0.9881			0.3768				0.009	−1.7279			−0.2483		
**Part 3**															
**Model Summary (y: aoPWV_Radial_SCOR; x: Sex; w: Age): R = 0.5356; R^2^ = 0.2869; *p* = <0.0001**															
			Coeff			SE	p					95% CI LL			95% CI UL
Intercept			5.7863			0.0808	0.000					5.6277			5.945
Sex (Female: 1; Male: 0)			0.2259			0.1176	0.055					−0.0049			0.4567
Age (years)			0.0362			0.0026	<0.0001					0.0311			0.0412
Sex * Age			−0.0059			0.0038	0.121					−0.0134			0.0016
**aoPWV_Carotid_SCOR (m/s)**															
**Model Summary (y: aoPWV_Carotid_SCOR; x: Sex; w: Age): R = 0.2791; R^2^ = 0.0779; *p* = <0.0001**															
			Coeff			SE	p					95% CI LL			95% CI UL
Intercept			6.3469			0.0937	0.000					6.1629			6.5308
Sex (Female: 1; Male: 0)			−0.1391			0.1356	0.306					−0.4055			0.1273
Age (years)			0.0139			0.0028	<0.0001					0.0085			0.0194
Sex * Age			0.0009			0.0041	0.823					−0.0072			0.009
**aoPWV_Brachial_MOG (m/s))**															
**Model Summary (y: aoPWV_Brachial_MOG; x: Sex; w: Age): R = 0.8928; R^2^ = 0.797; *p* = <0.0001**															
			Coeff			SE	p					95% CI LL			95% CI UL
Intercept			3.7984			0.042	<0.0001					3.7158			3.8811
Sex (Female: 1; Male: 0)			−0.262			0.058	<0.0001					−0.376			−0.1481
Age (years)			0.0715			0.002	<0.0001					0.0676			0.0754
Sex * Age			0.0034			0.003	0.2074					−0.002			0.0086

CI: confidence interval. LL and UL: lower and upper limit. Coeff: coefficient. SE: standard error.

**Table 8:** cfPWV _Real_SCOR levels and related parameters (CAVI and CAVIo) reference intervals: All.

	cfPWV_Real_SCOR (m/s)						CAVI_cfPWV_Real_SCOR						CAVIo_cfPWV_Real_SCOR					
Age (y)	50th	75th	90th	95th	97.5th	99th	50th	75th	90th	95th	97.5th	99th	50th	75th	90th	95th	97.5th	99th
5	4.6	5.1	5.5	5.8	6.1	6.5	4.5	5.5	6.7	7.5	8.3	9.3	6.4	7.8	9.2	10.3	11.3	12.6
10	5.0	5.5	6.0	6.3	6.6	7.0	4.8	5.7	6.8	7.6	8.4	9.4	7.1	8.5	10.0	11.1	12.2	13.6
15	5.6	6.1	6.7	7.0	7.4	7.8	5.7	6.8	8.0	8.9	9.7	10.8	8.4	10.0	11.7	12.9	14.1	15.6
20	6.2	6.8	7.4	7.8	8.1	8.5	6.8	8.1	9.4	10.4	11.3	12.4	9.8	11.5	13.4	14.8	16.1	17.7
25	6.7	7.4	8.0	8.4	8.8	9.2	7.9	9.2	10.8	11.8	12.8	14.1	11.0	12.9	15.0	16.5	17.8	19.6
30	7.2	7.8	8.5	8.9	9.3	9.8	8.7	10.3	11.9	13.0	14.1	15.5	12.0	14.1	16.3	17.8	19.3	21.2
35	7.5	8.2	8.9	9.3	9.7	10.2	9.4	11.0	12.8	14.0	15.2	16.7	12.7	14.9	17.3	18.9	20.5	22.5
40	7.7	8.4	9.2	9.6	10.1	10.6	9.8	11.6	13.5	14.8	16.1	17.8	13.2	15.5	18.0	19.8	21.4	23.6
45	7.8	8.6	9.4	9.9	10.4	11.0	10.2	12.1	14.1	15.5	16.9	18.7	13.6	16.0	18.7	20.5	22.3	24.6
50	7.9	8.8	9.6	10.2	10.7	11.3	10.5	12.5	14.7	16.2	17.7	19.7	13.9	16.5	19.4	21.4	23.3	25.8
55	8.1	9.0	9.8	10.4	11.0	11.7	10.8	13.0	15.4	17.1	18.8	20.9	14.4	17.2	20.4	22.6	24.7	27.5
60	8.3	9.2	10.2	10.8	11.4	12.2	11.4	13.8	16.5	18.4	20.3	22.7	15.2	18.4	21.9	24.4	26.8	30.0
65	8.6	9.6	10.7	11.4	12.0	12.8	12.4	15.1	18.2	20.4	22.6	25.4	16.6	20.2	24.3	27.2	30.1	33.9
70	9.1	10.3	11.4	12.1	12.8	13.7	14.1	17.3	21.0	23.7	26.3	29.8	19.0	23.3	28.3	31.8	35.4	40.1
75	10.0	11.2	12.5	13.3	14.1	15.1	17.0	21.0	25.7	29.1	32.5	36.9	23.0	28.6	34.9	39.6	44.2	50.4
80	11.3	12.7	14.1	15.0	15.9	17.0	22.0	27.6	33.9	38.6	43.2	49.4	30.2	37.8	46.7	53.2	59.7	68.5

**Table 9:** crPWV_SCOR levels and related parameters (CAVI and CAVIo) reference intervals: All.

	crPWV_SCOR (m/s)						CAVI_crPWV_SCOR						CAVIo_crPWV_SCOR					
Age (y)	50th	75th	90th	95th	97.5th	99th	50th	75th	90th	95th	97.5th	99th	50th	75th	90th	95th	97.5th	99th
5	7.0	7.9	8.9	9.5	10.0	10.7	10.5	13.4	16.5	18.7	20.7	23.2	14.4	18.1	22.1	24.7	27.2	30.3
10	7.7	8.6	9.5	10.0	10.5	11.2	11.6	14.4	17.4	19.3	21.2	23.5	16.4	20.2	24.0	26.6	29.0	32.0
15	8.2	9.1	10.0	10.5	11.0	11.6	12.6	15.4	18.3	20.2	22.0	24.2	17.8	21.6	25.5	28.0	30.4	33.3
20	8.6	9.5	10.4	10.9	11.4	12.0	13.4	16.2	19.1	21.1	22.8	25.1	19.0	22.8	26.6	29.2	31.6	34.5
25	8.9	9.8	10.7	11.3	11.8	12.4	14.1	17.0	20.0	21.9	23.7	26.0	19.9	23.7	27.7	30.2	32.6	35.6
30	9.2	10.2	11.1	11.6	12.2	12.8	14.9	17.8	20.8	22.8	24.6	26.9	20.7	24.6	28.6	31.2	33.6	36.5
35	9.5	10.5	11.4	12.0	12.5	13.1	15.5	18.6	21.6	23.7	25.5	27.9	21.5	25.4	29.4	32.1	34.5	37.5
40	9.7	10.7	11.7	12.3	12.8	13.5	16.2	19.3	22.4	24.5	26.4	28.8	22.2	26.2	30.2	32.9	35.3	38.3
45	10.0	11.0	12.0	12.6	13.1	13.8	16.8	20.0	23.2	25.4	27.3	29.7	22.8	26.9	31.0	33.7	36.1	39.2
50	10.2	11.2	12.3	12.9	13.5	14.1	17.4	20.7	24.0	26.2	28.2	30.7	23.4	27.5	31.7	34.4	36.9	40.0
55	10.4	11.5	12.5	13.2	13.8	14.5	18.0	21.4	24.8	27.0	29.1	31.7	23.9	28.1	32.4	35.1	37.7	40.8
60	10.6	11.7	12.8	13.4	14.1	14.8	18.6	22.1	25.6	27.9	30.0	32.6	24.5	28.7	33.0	35.8	38.4	41.6
65	10.8	11.9	13.0	13.7	14.3	15.1	19.2	22.7	26.3	28.7	30.9	33.6	25.0	29.3	33.7	36.5	39.1	42.3
70	11.0	12.1	13.3	14.0	14.6	15.4	19.7	23.4	27.1	29.5	31.7	34.5	25.5	29.8	34.3	37.1	39.8	43.0
75	11.1	12.3	13.5	14.2	14.9	15.7	20.3	24.0	27.8	30.3	32.6	35.5	25.9	30.4	34.9	37.8	40.4	43.7
80	11.3	12.5	13.7	14.5	15.2	16.0	20.8	24.7	28.6	31.1	33.5	36.4	26.4	30.9	35.4	38.4	41.1	44.4

**Table 10:** aoPWV_Radial_SCOR levels and related parameters (CAVI and CAVIo) reference intervals: All.

	aoPWV_Radial_SCOR (m/s)						CAVI_aoPWV_Radial_SCOR						CAVIo_aoPWV_Radial_SCOR					
Age (y)	50th	75th	90th	95th	97.5th	99th	50th	75th	90th	95th	97.5th	99th	50th	75th	90th	95th	97.5th	99th
5	4.9	5.4	5.9	6.2	6.5	6.8	5.1	6.2	7.3	8.0	8.7	9.6	7.4	8.9	10.5	11.6	12.6	13.9
10	6.0	6.6	7.1	7.4	7.7	8.0	6.9	8.2	9.6	10.5	11.3	12.4	10.0	12.0	14.1	15.5	16.8	18.4
15	6.6	7.1	7.7	8.0	8.2	8.6	8.0	9.4	10.9	11.9	12.8	13.9	11.4	13.7	16.0	17.6	19.0	20.9
20	6.9	7.5	8.0	8.3	8.6	8.9	8.6	10.1	11.7	12.7	13.7	14.9	12.3	14.6	17.1	18.8	20.3	22.2
25	7.2	7.7	8.2	8.6	8.8	9.1	9.0	10.6	12.2	13.3	14.3	15.5	12.8	15.2	17.7	19.4	21.0	23.0
30	7.3	7.9	8.4	8.7	9.0	9.3	9.3	10.9	12.6	13.7	14.7	15.9	13.1	15.5	18.1	19.8	21.4	23.4
35	7.4	8.0	8.5	8.8	9.1	9.4	9.5	11.1	12.8	13.9	14.9	16.2	13.2	15.7	18.2	19.9	21.5	23.5
40	7.5	8.1	8.6	8.9	9.2	9.5	9.6	11.2	12.9	14.0	15.0	16.3	13.3	15.7	18.2	19.9	21.5	23.5
45	7.6	8.1	8.6	8.9	9.2	9.5	9.6	11.3	13.0	14.1	15.1	16.4	13.2	15.6	18.2	19.8	21.4	23.3
50	7.6	8.1	8.7	9.0	9.2	9.5	9.6	11.3	13.0	14.1	15.1	16.4	13.1	15.5	18.0	19.6	21.2	23.1
55	7.6	8.2	8.7	9.0	9.2	9.5	9.6	11.3	13.0	14.1	15.1	16.4	13.0	15.3	17.8	19.4	20.9	22.7
60	7.6	8.2	8.7	9.0	9.2	9.5	9.6	11.2	12.9	14.0	15.0	16.3	12.8	15.1	17.5	19.1	20.5	22.4
65	7.6	8.2	8.7	9.0	9.2	9.5	9.5	11.2	12.8	13.9	15.0	16.2	12.7	14.9	17.2	18.8	20.2	22.0
70	7.6	8.2	8.6	8.9	9.2	9.5	9.4	11.1	12.7	13.8	14.9	16.1	12.5	14.7	16.9	18.4	19.8	21.5
75	7.6	8.1	8.6	8.9	9.2	9.5	9.3	11.0	12.6	13.7	14.7	16.0	12.2	14.4	16.6	18.1	19.4	21.1
80	7.6	8.1	8.6	8.9	9.1	9.4	9.2	10.9	12.5	13.6	14.6	15.8	12.0	14.1	16.3	17.7	19.0	20.7

**Table 11:** aoPWV_Carotid_SCOR levels and related parameters (CAVI and CAVIo) reference intervals: All.

	aoPWV_Carotid_SCOR (m/s)						CAVI_aoPWV_Carotid_SCOR						CAVIo_aoPWV_Carotid_SCOR					
Age (y)	50th	75th	90th	95th	97.5th	99th	50th	75th	90th	95th	97.5th	99th	50th	75th	90th	95th	97.5th	99th
5	5.9	6.7	7.4	7.8	8.2	8.7	7.0	8.8	11.0	12.5	14.1	16.2	10.5	13.3	16.4	18.6	20.7	23.5
10	6.2	6.9	7.5	7.9	8.2	8.6	7.3	8.9	10.7	12.0	13.2	14.9	10.7	13.1	15.7	17.4	19.2	21.4
15	6.4	7.0	7.6	7.9	8.2	8.6	7.5	9.0	10.6	11.7	12.8	14.1	10.8	12.9	15.3	16.8	18.3	20.2
20	6.6	7.1	7.6	7.9	8.2	8.6	7.7	9.0	10.5	11.5	12.4	13.7	10.9	12.9	15.0	16.4	17.7	19.4
25	6.7	7.2	7.7	8.0	8.2	8.5	7.8	9.0	10.4	11.3	12.2	13.3	10.9	12.8	14.8	16.1	17.3	18.8
30	6.7	7.2	7.7	8.0	8.2	8.5	7.9	9.1	10.3	11.2	12.0	13.0	11.0	12.8	14.6	15.8	16.9	18.4
35	6.8	7.3	7.7	8.0	8.2	8.5	7.9	9.1	10.3	11.1	11.8	12.8	11.0	12.7	14.4	15.6	16.6	18.0
40	6.9	7.3	7.7	8.0	8.2	8.5	8.0	9.1	10.2	11.0	11.7	12.6	11.1	12.7	14.3	15.4	16.4	17.6
45	6.9	7.4	7.8	8.0	8.2	8.5	8.1	9.1	10.2	10.9	11.6	12.4	11.1	12.6	14.2	15.2	16.2	17.4
50	7.0	7.4	7.8	8.0	8.2	8.5	8.1	9.1	10.2	10.8	11.5	12.2	11.1	12.6	14.1	15.1	16.0	17.1
55	7.0	7.4	7.8	8.0	8.2	8.5	8.2	9.1	10.1	10.8	11.4	12.1	11.2	12.6	14.0	15.0	15.8	16.9
60	7.1	7.5	7.8	8.0	8.2	8.4	8.2	9.2	10.1	10.7	11.3	12.0	11.2	12.6	13.9	14.8	15.7	16.7
65	7.1	7.5	7.8	8.0	8.2	8.4	8.3	9.2	10.1	10.7	11.2	11.8	11.2	12.5	13.9	14.7	15.5	16.5
70	7.2	7.5	7.8	8.0	8.2	8.4	8.3	9.2	10.0	10.6	11.1	11.7	11.2	12.5	13.8	14.6	15.4	16.3
75	7.2	7.5	7.9	8.1	8.2	8.4	8.4	9.2	10.0	10.6	11.0	11.6	11.3	12.5	13.7	14.5	15.3	16.2
80	7.2	7.6	7.9	8.1	8.2	8.4	8.4	9.2	10.0	10.5	11.0	11.6	11.3	12.5	13.7	14.5	15.2	16.0

**Table 12:** aoPWV_Brachial_MOG levels and related parameters (CAVI and CAVIo) reference intervals: All.

	aoPWV_Brachial_MOG (m/s)						CAVI_aoPWV_Brachial_MOG						CAVIo_aoPWV_Brachial_MOG					
Age (y)	50th	75th	90th	95th	97.5th	99th	50th	75th	90th	95th	97.5th	99th	50th	75th	90th	95th	97.5th	99th
5	4.0	4.1	4.2	4.3	4.4	4.5	3.3	3.5	3.6	3.7	3.8	3.9	4.9	5.3	5.7	5.9	6.2	6.4
10	4.5	4.7	5.0	5.1	5.3	5.4	3.7	4.1	4.4	4.6	4.8	5.1	5.5	6.2	6.8	7.3	7.6	8.1
15	4.8	5.1	5.4	5.5	5.7	5.9	4.1	4.6	5.1	5.4	5.7	6.0	6.0	6.8	7.7	8.3	8.8	9.5
20	5.0	5.4	5.7	5.8	6.0	6.2	4.5	5.1	5.7	6.0	6.4	6.8	6.4	7.4	8.5	9.2	9.8	10.6
25	5.3	5.6	6.0	6.2	6.3	6.6	4.9	5.5	6.2	6.6	7.1	7.6	6.8	8.0	9.1	9.9	10.6	11.5
30	5.6	5.9	6.3	6.5	6.7	6.9	5.3	6.0	6.7	7.2	7.7	8.2	7.3	8.5	9.7	10.6	11.4	12.3
35	5.9	6.3	6.6	6.9	7.1	7.3	5.7	6.5	7.3	7.8	8.3	8.9	7.8	9.1	10.4	11.3	12.1	13.1
40	6.2	6.6	7.0	7.3	7.5	7.8	6.3	7.1	8.0	8.5	9.1	9.7	8.4	9.7	11.1	12.0	12.9	13.9
45	6.6	7.0	7.5	7.8	8.0	8.3	6.9	7.8	8.8	9.4	10.0	10.7	9.2	10.6	12.0	12.9	13.8	14.9
50	7.0	7.5	8.0	8.3	8.6	9.0	7.8	8.8	9.8	10.5	11.1	11.9	10.2	11.6	13.1	14.1	15.0	16.1
55	7.4	8.0	8.6	8.9	9.3	9.7	8.8	10.0	11.2	11.9	12.6	13.5	11.5	13.0	14.6	15.6	16.5	17.7
60	7.9	8.6	9.2	9.7	10.0	10.5	10.3	11.6	13.0	13.9	14.7	15.8	13.3	14.9	16.6	17.7	18.7	20.0
65	8.4	9.2	10.0	10.5	10.9	11.4	12.2	13.9	15.6	16.7	17.8	19.1	15.7	17.6	19.4	20.7	21.8	23.2
70	9.0	9.9	10.8	11.4	11.9	12.5	14.8	17.0	19.2	20.8	22.2	23.9	19.0	21.3	23.5	24.9	26.2	27.9
75	9.6	10.7	11.7	12.4	13.0	13.8	18.4	21.4	24.6	26.8	28.8	31.3	23.9	26.6	29.4	31.2	32.9	34.9
80	10.3	11.5	12.8	13.6	14.3	15.2	23.5	28.0	32.8	36.1	39.2	43.2	30.9	34.6	38.3	40.7	43.0	45.7

**Table 13:** PWV Ratio levels and related parameters (CAVI and CAVIo) reference intervals: All.

	PWV_Ratio						CAVI_PWV_Ratio						CAVIo_PWV_Ratio					
Age (y)	50th	75th	90th	95th	97.5th	99th	50th	75th	90th	95th	97.5th	99th	50th	75th	90th	95th	97.5th	99th
5	0.58	0.67	0.76	0.82	0.88	0.95	0.33	0.44	0.57	0.67	0.76	0.89	0.35	0.46	0.59	0.68	0.78	0.90
10	0.64	0.73	0.82	0.87	0.93	0.99	0.41	0.53	0.66	0.76	0.85	0.98	0.43	0.54	0.68	0.77	0.87	0.99
15	0.69	0.77	0.85	0.90	0.95	1.01	0.47	0.59	0.72	0.82	0.91	1.03	0.48	0.60	0.74	0.83	0.92	1.04
20	0.71	0.79	0.87	0.92	0.97	1.03	0.51	0.63	0.76	0.86	0.95	1.06	0.52	0.64	0.77	0.86	0.95	1.07
25	0.73	0.81	0.89	0.93	0.98	1.03	0.53	0.66	0.79	0.88	0.97	1.08	0.54	0.66	0.80	0.89	0.97	1.08
30	0.74	0.82	0.89	0.94	0.98	1.04	0.55	0.67	0.80	0.89	0.98	1.09	0.56	0.68	0.81	0.90	0.98	1.09
35	0.75	0.82	0.90	0.95	0.99	1.04	0.56	0.68	0.81	0.90	0.99	1.10	0.57	0.69	0.82	0.91	0.99	1.10
40	0.75	0.83	0.91	0.95	1.00	1.05	0.57	0.69	0.83	0.92	1.01	1.12	0.58	0.70	0.83	0.92	1.01	1.12
45	0.76	0.84	0.92	0.96	1.01	1.07	0.58	0.71	0.84	0.94	1.03	1.15	0.59	0.71	0.85	0.95	1.04	1.15
50	0.77	0.85	0.93	0.98	1.03	1.09	0.59	0.73	0.87	0.98	1.07	1.20	0.60	0.74	0.88	0.98	1.08	1.20
55	0.78	0.87	0.96	1.01	1.06	1.13	0.62	0.76	0.92	1.03	1.14	1.28	0.63	0.77	0.93	1.04	1.15	1.28
60	0.81	0.90	1.00	1.06	1.11	1.18	0.66	0.82	1.00	1.12	1.24	1.40	0.67	0.83	1.01	1.13	1.25	1.41
65	0.85	0.95	1.05	1.12	1.18	1.25	0.72	0.90	1.11	1.25	1.39	1.58	0.73	0.92	1.12	1.27	1.41	1.59
70	0.90	1.01	1.13	1.20	1.27	1.36	0.82	1.03	1.28	1.45	1.62	1.84	0.83	1.05	1.29	1.47	1.64	1.86
75	0.98	1.11	1.24	1.32	1.40	1.50	0.96	1.23	1.53	1.75	1.96	2.24	0.97	1.24	1.55	1.77	1.98	2.27
80	1.08	1.23	1.39	1.49	1.58	1.69	1.19	1.53	1.92	2.20	2.48	2.84	1.19	1.54	1.94	2.23	2.51	2.89

## Data Availability

The data presented in thos study are available within the article and in supplementary material.

## Supplementary Materials

The following are available online https://www.mdpi.com/2308-3425/8/1/3/s1. Figure S1. Age-related profiles for cfPWV_Real_SCOR for the RIs group. Figure S2. Age-related profiles for CAVI_cfPWV_Real_SCOR for the RIs group.Figure S3. Age-related profiles for CAVIo_cfPWV_Real_SCOR for the RIs group. Figure S4. Age-related profiles for crPWV_SCOR for the RIs group. Figure S5. Age-related profiles for CAVI_crPWV_SCOR for the RIs group. Figure S6. Age-related profiles for CAVIo_crPWV_SCOR, for the RIs group. Figure S7. Age-related profiles for aoPWV_Radial_SCOR, for the RIs group. Figure S8. Age-related profiles for CAVI_aoPWV_Radial_SCOR for the RIs group. Figure S9. Age-related profiles for CAVIo_aoPWV_Radial_SCOR for the RIs group. Figure S10. Age-related profiles for aoPWV_Carotid_SCOR for the RIs group. Figure S11. Age-related profiles for CAVI_aoPWV_Carotid_SCOR for the RIs group. Figure S12. Age-related profiles for CAVIo_aoPWV_Carotid_SCOR for the RIs group. Figure S13. Age-related profiles for aoPWV_Brachial_MOG for the RIs group. Figure S14. Age-related profiles for CAVI_aoPWV_Brachial_MOG for the RIs group. Figure S15. Age-related profiles for CAVIo_aoPWV_Brachial_MOG for the RIs group. Figure S16. Age-related profiles for PWV_Ratio for the RIs group. Figure S17. Age-related profiles for CAVI_PWV_Ratio for the RIs group. Figure S18. Age-related profiles for CAVIo_PWV_Ratio for the RIs group. Table S1. Subjects demographic, anthropometric, clinical and cardiovascular characteristics: All (*n* = 3169). Table S2. Subjects demographic, anthropometric, clinical and cardiovascular characteristics: Reference Intervals group (*n* = 1289). Table S3. Subjects demographic, anthropometric, clinical and cardiovascular characteristics: Excluded group (*n* = 2330). Table S4. Age-related mean value and standard deviation equations: mathematical model summary (Reference Intervals group). Table S5. cfPWV_Real_SCOR [m/s] reference intervals: All. Table S6. cfPWV_Real_SCOR [m/s] reference intervals: Female. Table S7. cfPWV_Real_SCOR [m/s] reference intervals: Male. Table S8. CAVI_cfPWV_Real_SCOR [m/s] reference intervals: All. Table S9. CAVI_cfPWV_Real_SCOR [m/s] reference intervals: Females. Table S10. CAVI_cfPWV_Real_SCOR [m/s] reference intervals: Males. Table S11. CAVIo_cfPWV_Real_SCOR [m/s] reference intervals: All. Table S12. CAVIo_cfPWV_Real_SCOR [m/s] reference intervals: Females. Table S13. CAVIo_cfPWV_Real_SCOR [m/s] reference intervals: Males. Table S14. crPWV_SCOR [m/s] reference intervals: All. Table S15. crPWV_SOR [m/s] reference intervals: Females. Table S16. crPWV_SCOR [m/s] reference intervals: Males. Table S17. CAVI_ crPWV_SCOR [m/s] reference intervals: All. Table S18. CAVI_crPWV_SCOR [m/s] reference intervals: Females. Table S19. CAVI_crPWV_SCOR [m/s] reference intervals: Males. Table S20. CAVIo_crPWV_SCOR [m/s] reference intervals: All. Table S21. CAVIo_crPWV_SCOR [m/s] reference intervals: Females. Table S22. CAVIo_crPWV_SCOR [m/s] reference intervals: Males. Table S23. aoPWV_Radial_SCOR [m/s] reference intervals: All. Table S24. aoPWV_Radial_SCOR [m/s] reference intervals: Females. Table S25. aoPWV_Radial_SCOR [m/s] reference intervals: Males. Table S26. CAVI_aoPWV_Radial_SCOR [m/s] reference intervals: All. Table S27. CAVI_aoPWV_Radial_SCOR [m/s] reference intervals: Females. Table S28. CAVI_aoPWV_Radial_SCOR [m/s] reference intervals: Males. Table S29. CAVIo_aoPWV_Radial_SCOR [m/s] reference intervals: All. Table S30. CAVIo_aoPWV_Radial_SCOR [m/s] reference intervals: Females. Table S31. CAVIo_aoPWV_Radial_SCOR [m/s] reference intervals: Males. Table S32. aoPWV_Carotid_SCOR [m/s] reference intervals: All. Table S33. aoPWV_Carotid_SCOR [m/s] reference intervals: Females. Table S34. aoPWV_Carotid_SCOR [m/s] reference intervals: Males. Table S35. CAVI_aoPWV_Carotid_SCOR [m/s] reference intervals: All. Table S36. CAVI_aoPWV_Carotid_SCOR [m/s] reference intervals: Females. Table S37. CAVI_aoPWV_Carotid_SCOR [m/s] reference intervals: Males. Table S38. CAVIo_aoPWV_Carotid_SCOR [m/s] reference intervals: All. Table S39. CAVIo_aoPWV_Carotid_SCOR [m/s] reference intervals: Females. Table S40. CAVIo_aoPWV_Carotid_SCOR [m/s] reference intervals: Males. Table S41. aoPWV_Brachial_MOG [m/s] reference intervals: All. Table S42. aoPWV_Brachial_MOG [m/s] reference intervals: Females. Table S43. aoPWV_Brachial_MOG [m/s] reference intervals: Males. Table S44. CAVI_aoPWV_Brachial_MOG [m/s] reference intervals: All. Table S45. CAVI_aoPWV_Brachial_MOG [m/s] reference intervals: Females. Table S46. CAVI_aoPWV_Brachial_MOG [m/s] reference intervals: Males. Table S47. CAVIo_aoPWV_Brachial_MOG [m/s] reference intervals: All. Table S48. CAVIo_aoPWV_Brachial_MOG [m/s] reference intervals: Females. Table S49. CAVIo_aoPWV_Brachial_MOG [m/s] reference intervals: Males. Table S50. PWV_Ratio reference intervals: All. Table S51. PWV_Ratio reference intervals: Females. Table S52. PWV_Ratio reference intervals: Males. Table S53. CAVI_PWV_Ratio reference intervals: All. Table S54. CAVI_PWV_Ratio reference intervals: Females. Table S55. CAVI_PWV_Ratio reference intervals: Males. Table S56. CAVIo_PWV_Ratio reference intervals: All. Table S57. CAVIo_PWV_Ratio reference intervals: Females. Table S58. CAVIo_PWV_Ratio reference intervals: Males.

## Abbreviations

**Abbreviation**	**Term**
aoPWV	Aortic pulse wave velocity
bBP	Brachial blood pressure
bDBP	Brachial diastolic blood pressure
BH	Body height
bMBP	Brachial mean blood pressure
BMI	Body mass index
BP	Blood pressure
bPP	Brachial pulse pressure
bSBP	Brachial systolic blood pressure
BW	Bodyweight
CAVI	Cardio-ankle vascular index
CCA	Common carotid artery
CCC	Concordance correlation coefficients
CFA	Common femoral artery
cfPWV	Carotid-femoral pulse wave velocity
CRFs	Cardiovascular risk factors
crPWV	Carotid-radial pulse wave velocity
CV	Cardiovascular
FPs	Fractional polynomials
GTF	General transfer function
HR	Heart rate
Ln	Natural logarithm
MOG	Mobil-O-Graph system or device
MV	Mean value
PWA	Pulse wave analysis
PWV	Pulse wave velocity
RIs	Reference intervals
SCOR	SphygmoCor system or device
SD	Standard deviation
WSA	Wave separation analysis
y	Years old
z-	Z score
β	Arterial stiffness index
β-PWV	Beta-PWV (or CAVI or CAVIo)
ρ	Blood mass density
