# Supplementary material for: Physiological Age- and Sex-Related Profiles for Local (Aortic) and Regional (Carotid-Femoral, Carotid-Radial) Pulse Wave Velocity and Center-to-Periphery Stiffness Gradient, with and without Blood Pressure Adjustments: Reference Intervals and Agreement between Methods in Healthy Subjects (3–84 Years)
Source: J Cardiovasc Dev Dis. 2021 Jan 12;8(1):3. doi: 10.3390/jcdd8010003 (PMC7827252; doi:10.3390/jcdd8010003)
Supplement: Supplementary file 1 [file jcdd-08-00003-s001.zip › jcdd-1029482-supplementary/jcdd-1029482-supplementary.pdf]

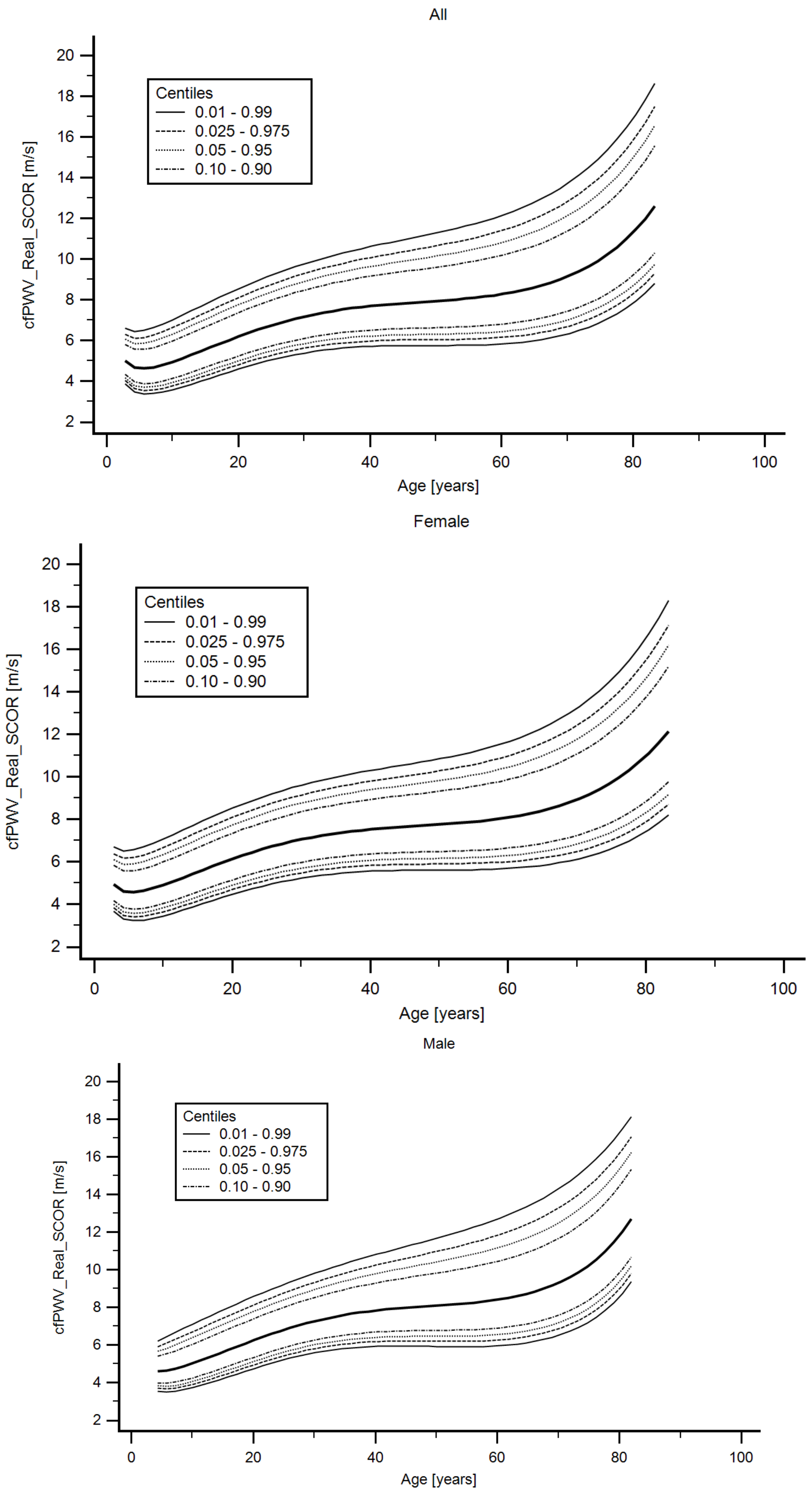

Figure S1. Age-related profiles for cfPWV_Real_SCOR, for subjects included in RIs group.

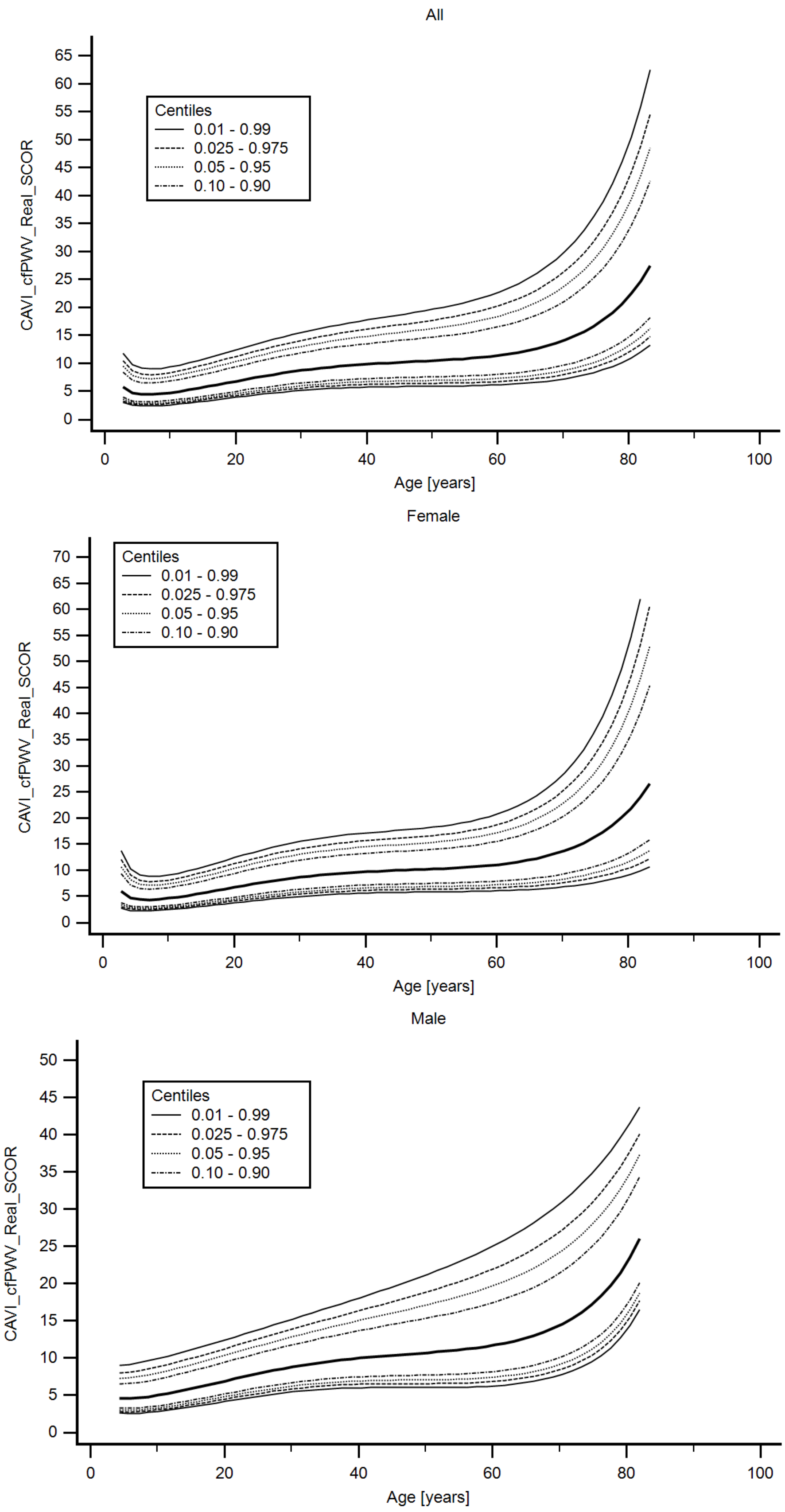

Figure S2. Age-related profiles for CAVI_cfPWV_Real_SCOR, for subjects included in RIs group.

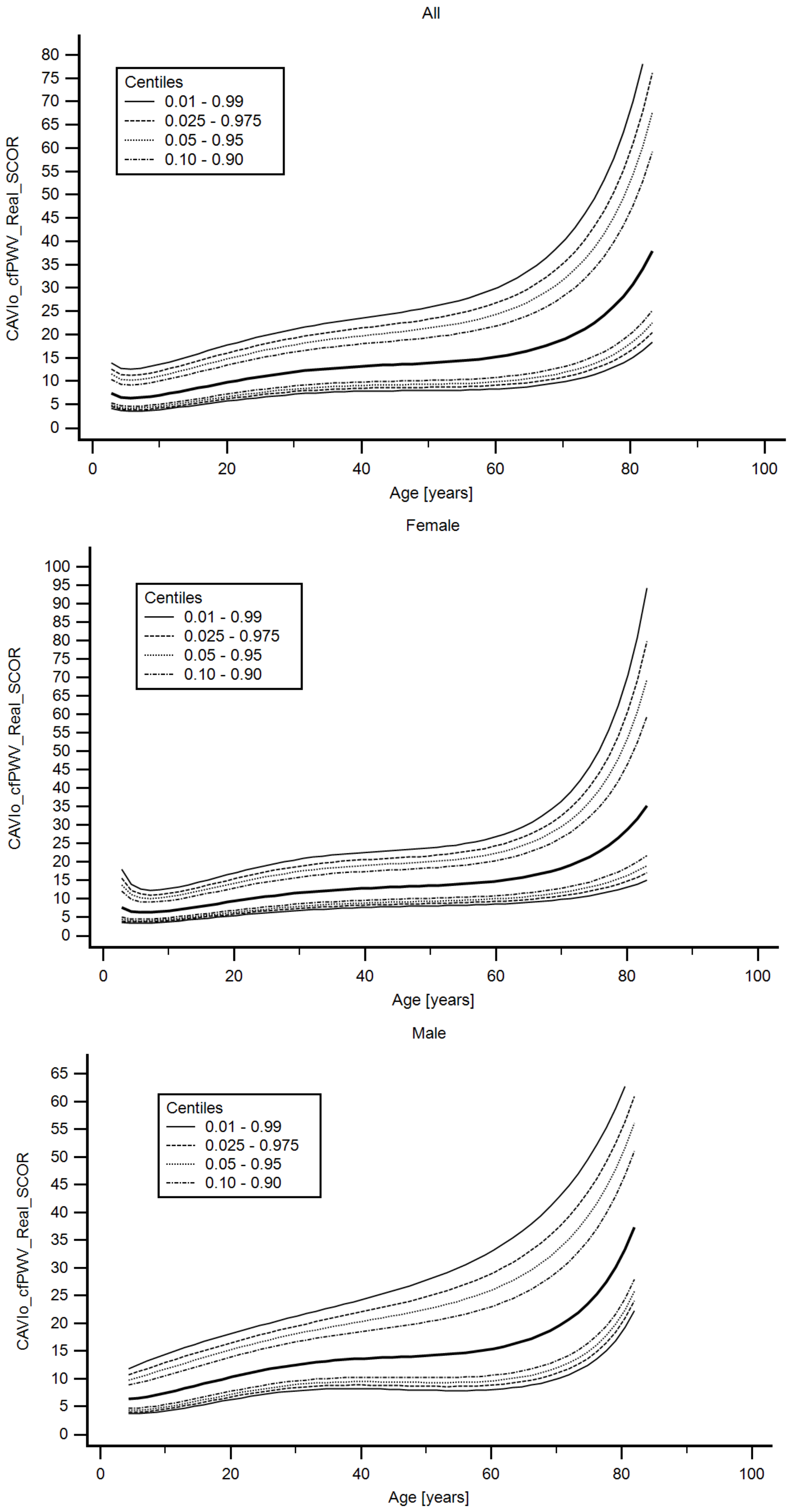

Figure S3. Age-related profiles for CAVIo_cfPWV_Real_SCOR, for subjects included in RIs group.

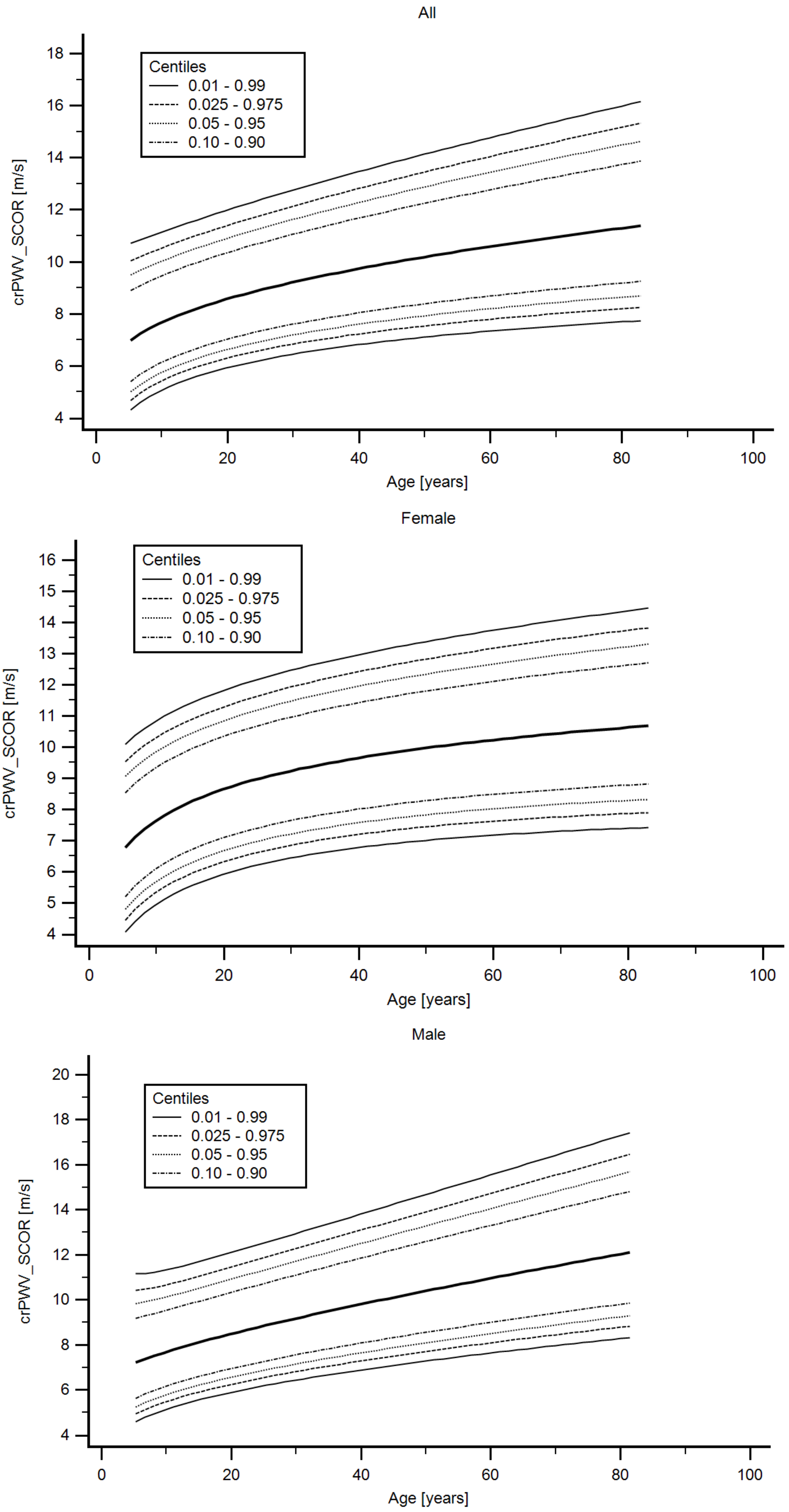

Figure S4. Age-related profiles for crPWV_SCOR, for subjects included in RIs group.

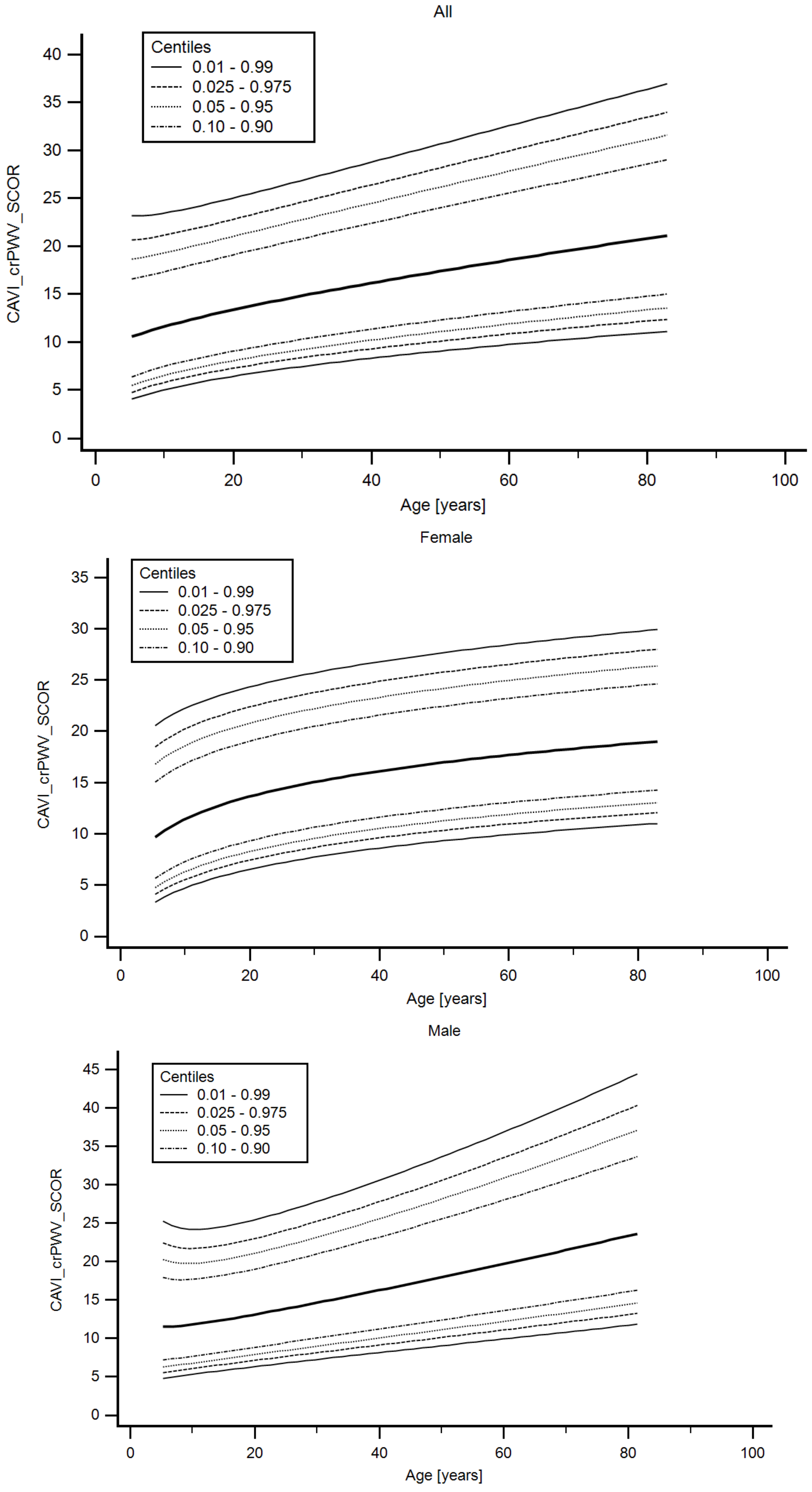

Figure S5. Age-related profiles for CAVI_crPWV_SCOR, for subjects included in RIs group.

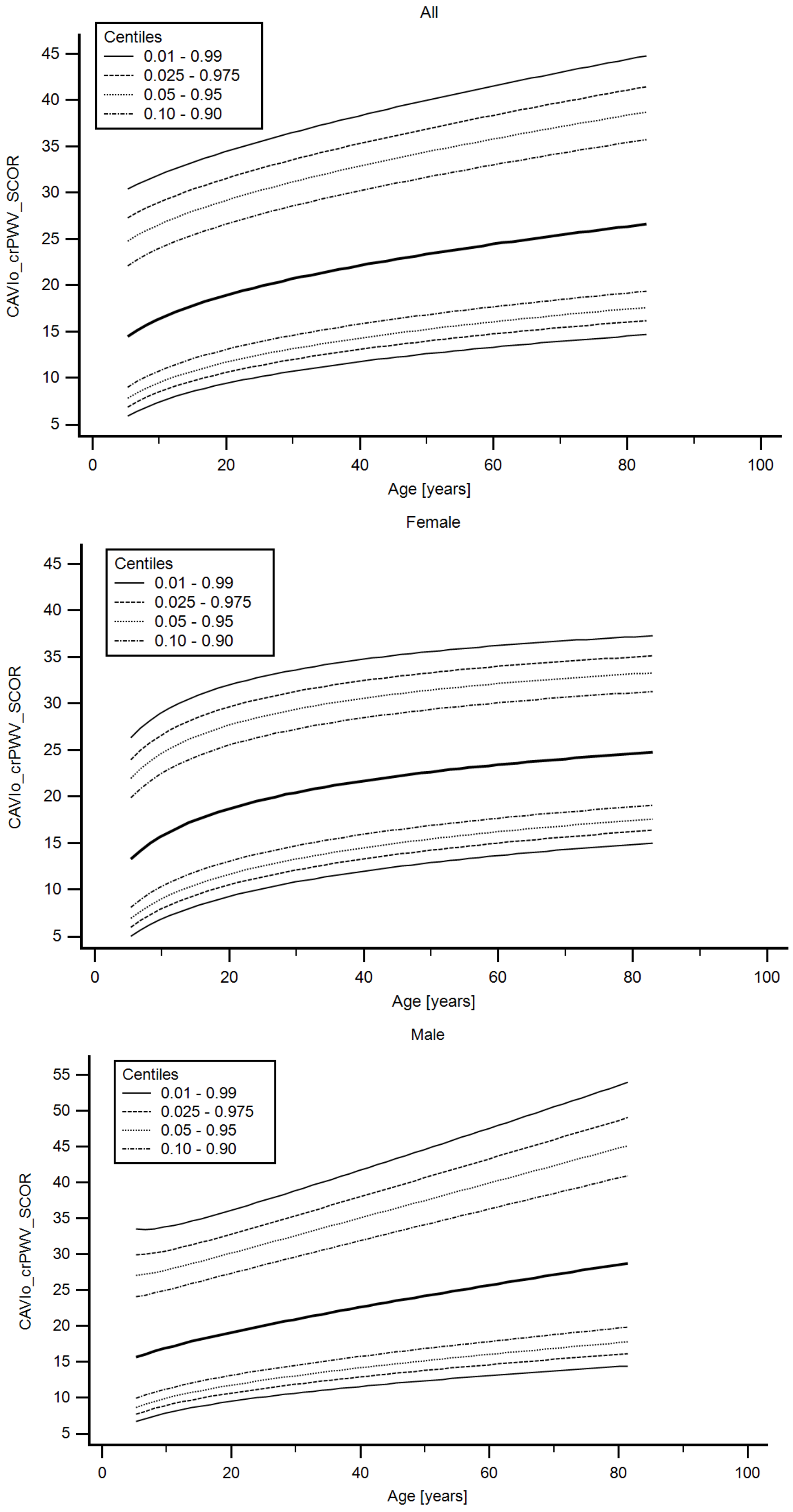

Figure S6. Age-related profiles for CAVIo_crPWV_SCOR, for subjects included in RIs group.

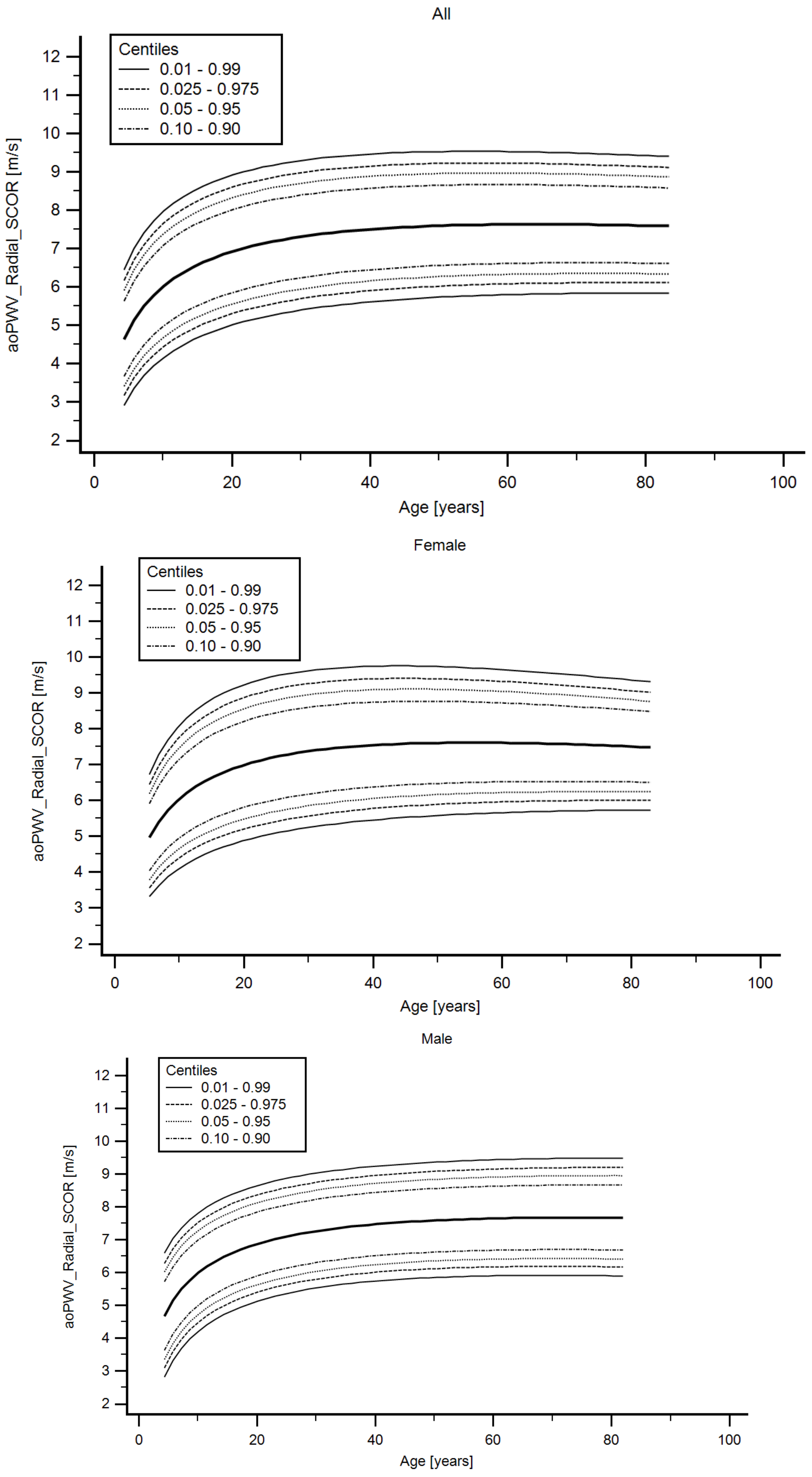

Figure S7. Age-related profiles for aoPWV_Radial_SCOR, for subjects included in RIs group.

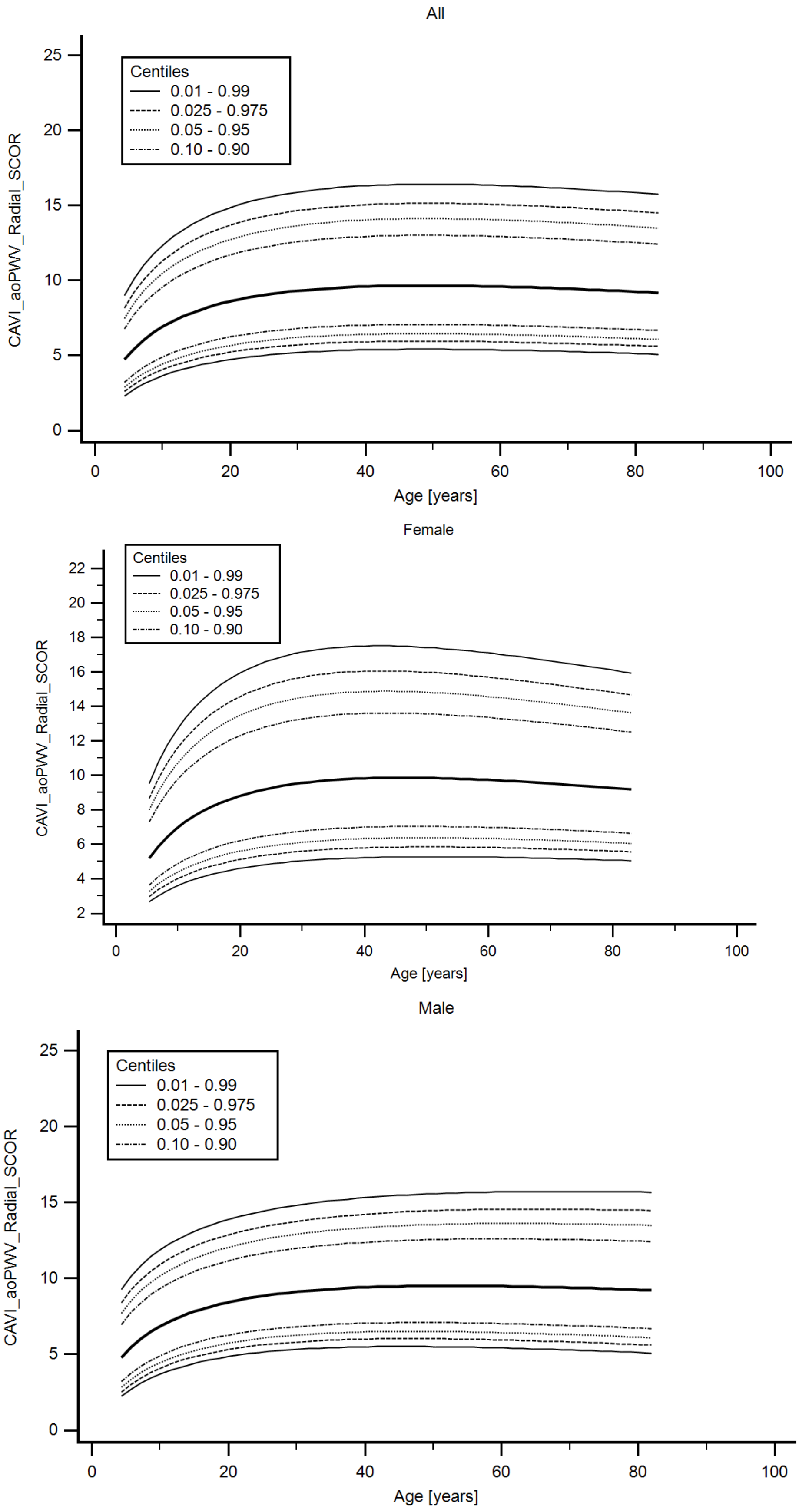

Figure S8. Age-related profiles for CAVI_aoPWV_Radial_SCOR, for subjects included in RIs group.

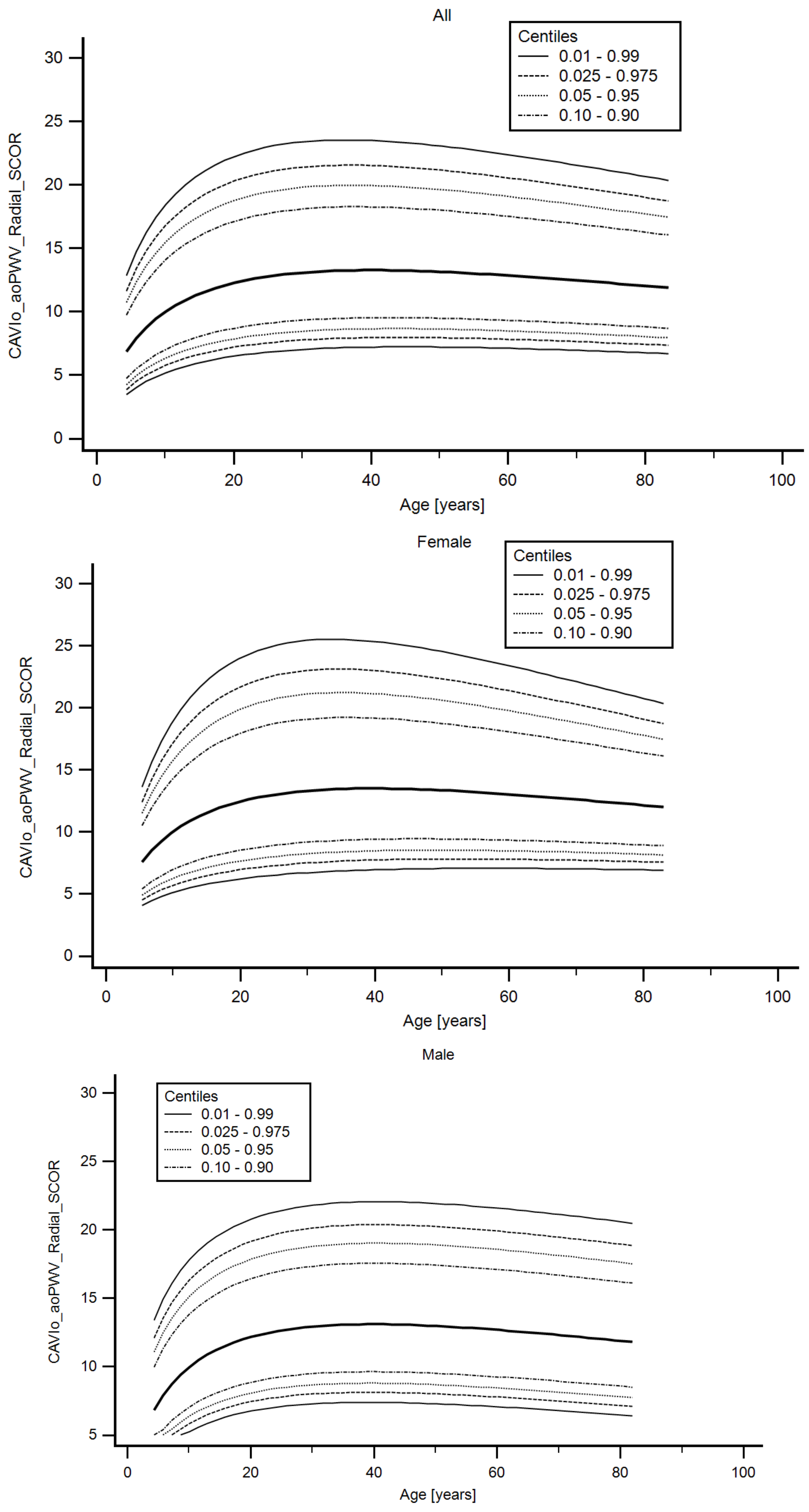

Figure S9. Age-related profiles for CAVIo_aoPWV_Radial_SCOR, for subjects included in RIs group.

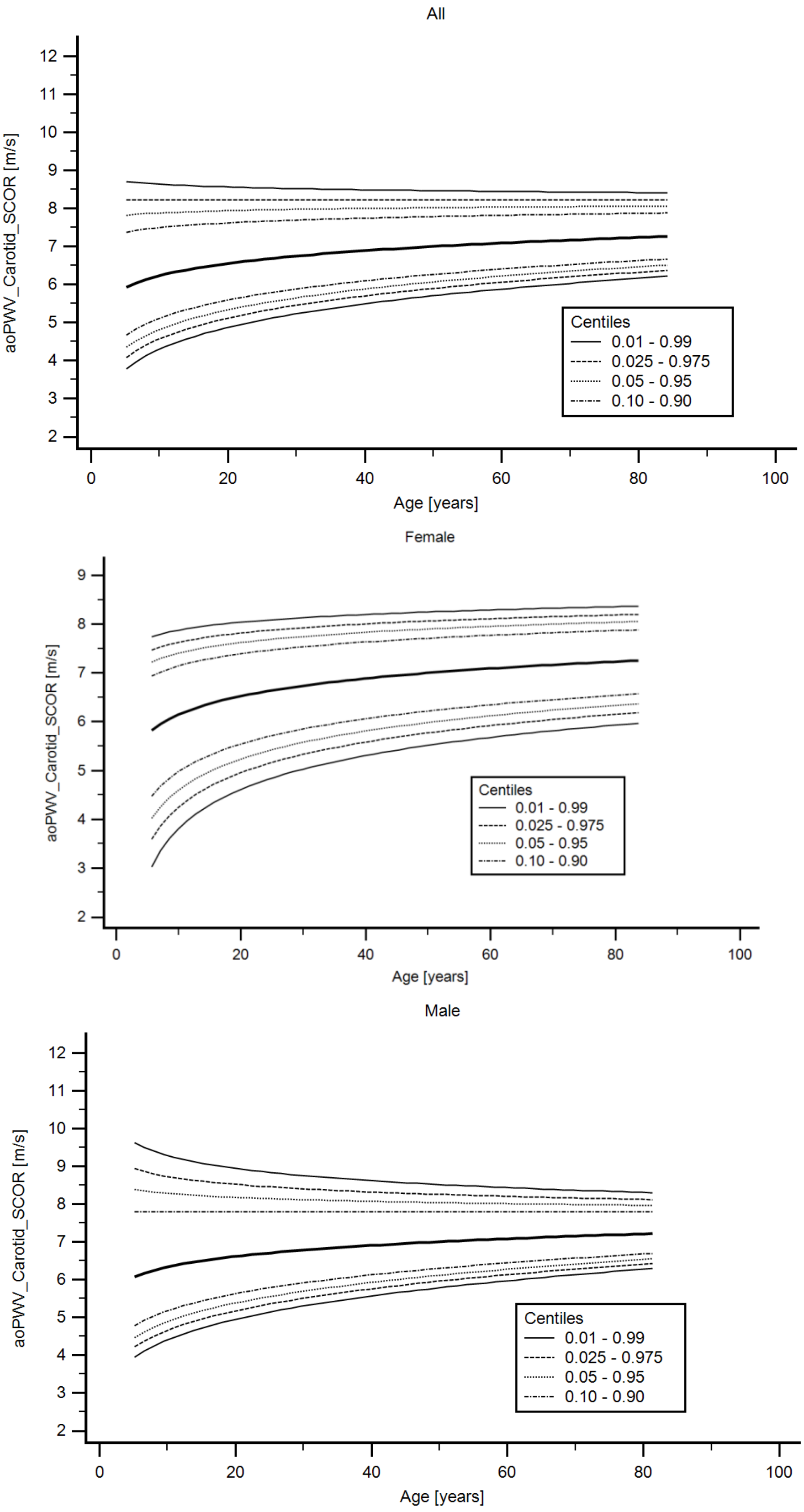

Figure S10. Age-related profiles for aoPWV_Carotid_SCOR, for subjects included in RIs group.

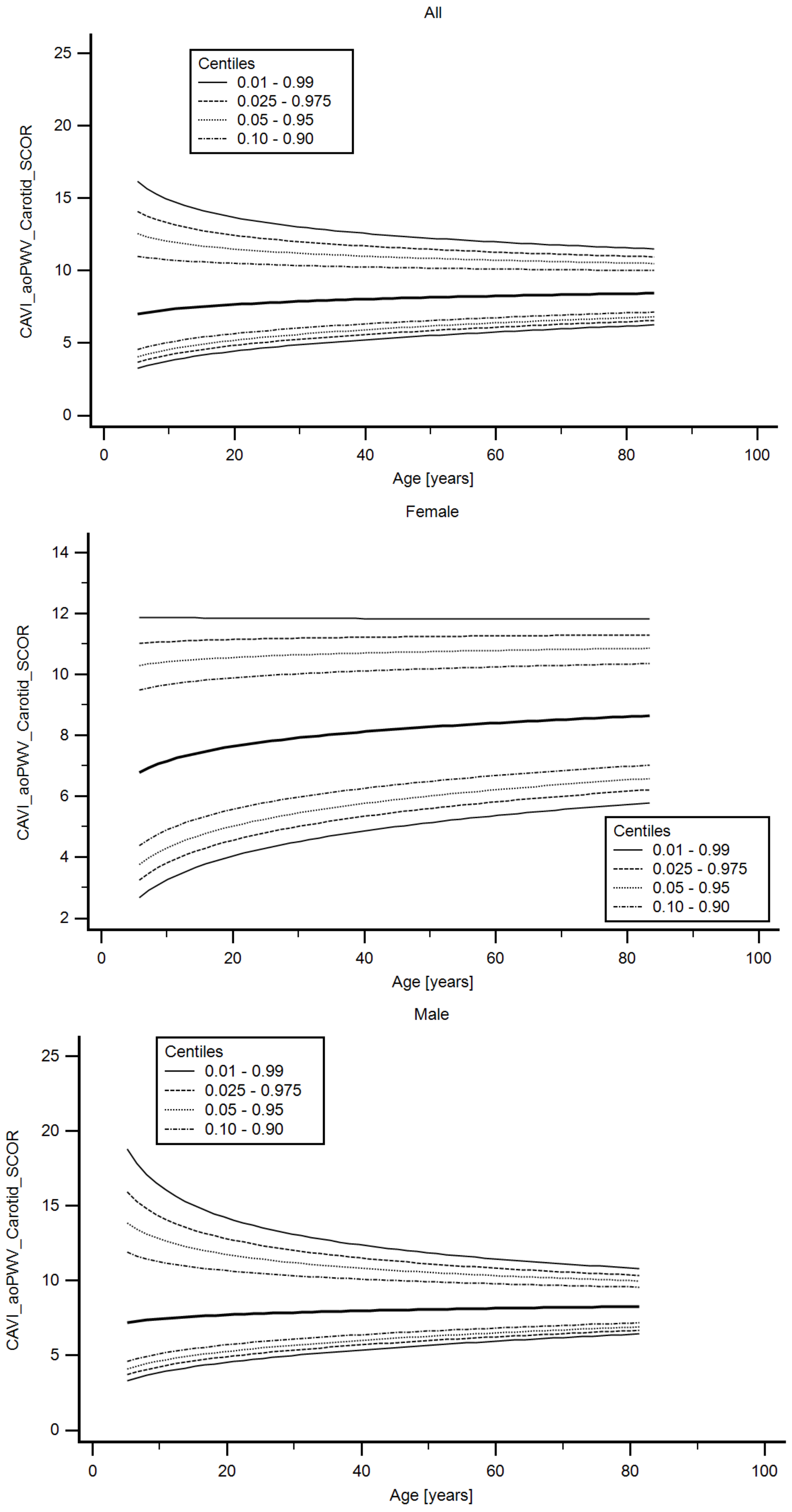

Figure S11. Age-related profiles for CAVI_aoPWV_Carotid_SCOR, for subjects included in RIs group.

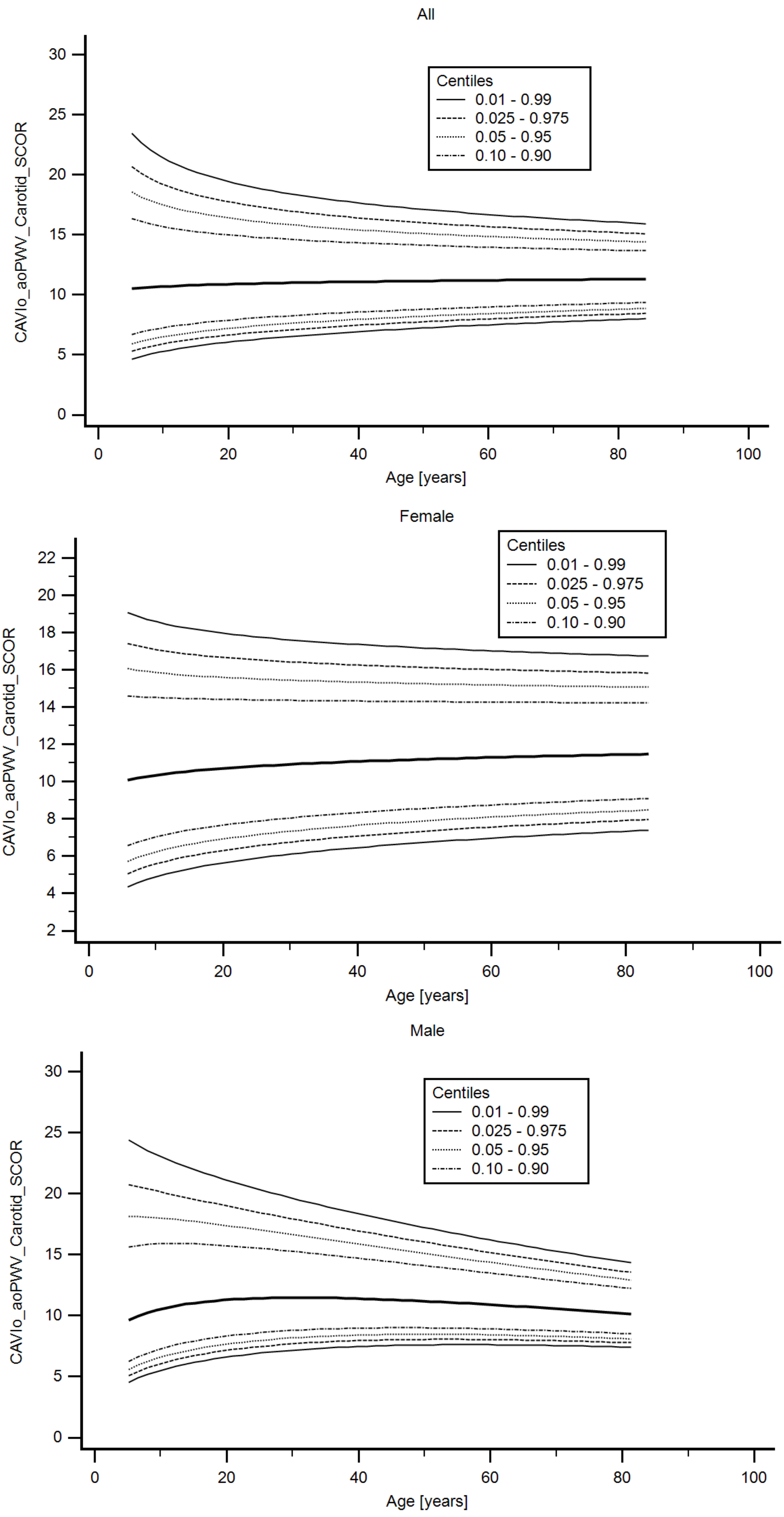

Figure S12. Age-related profiles for CAVIo_aoPWV_Carotid_SCOR, for subjects included in RIs group.

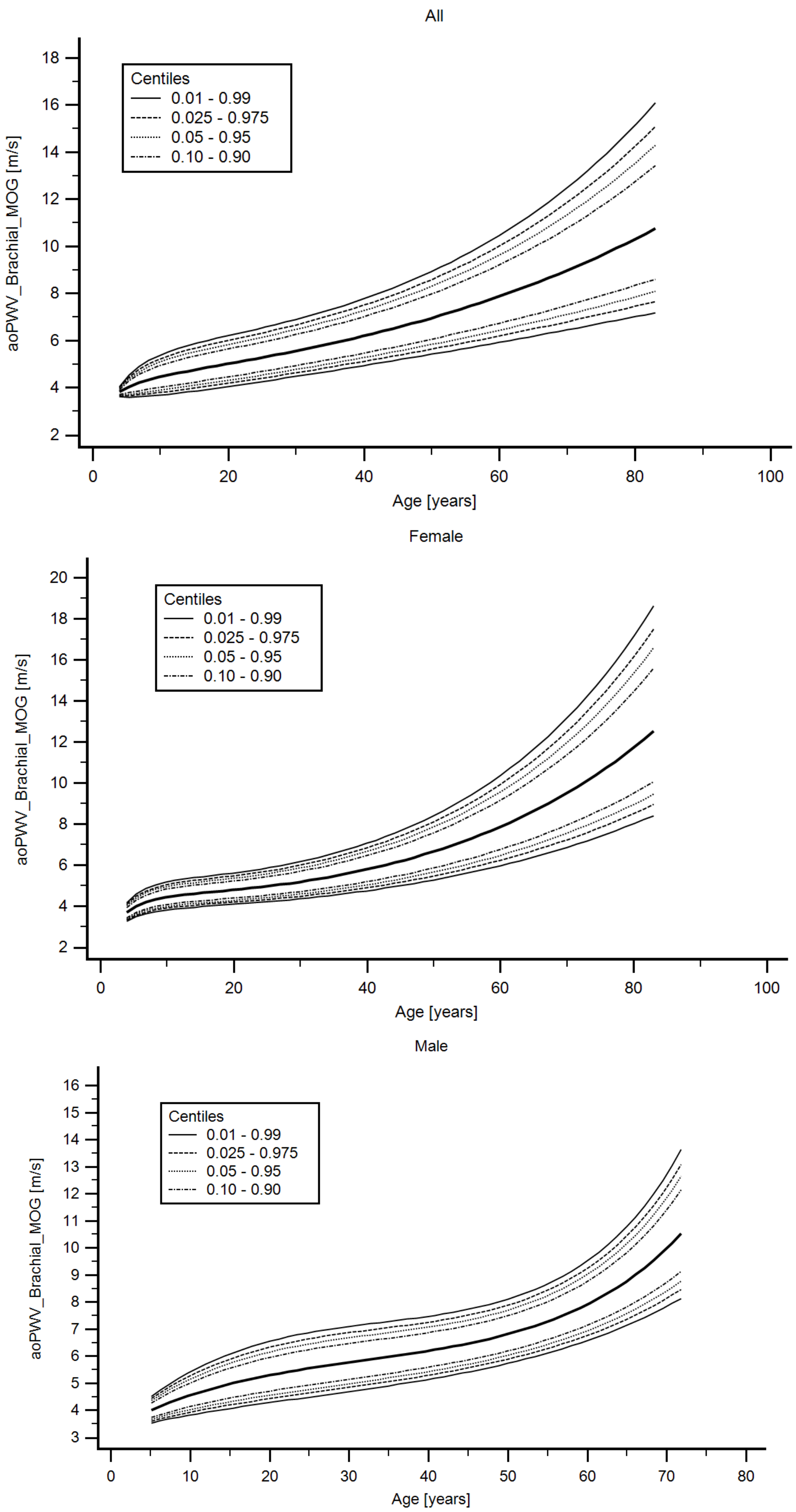

Figure S13. Age-related profiles for aoPWV_Brachial_MOG, for subjects included in RIs group.

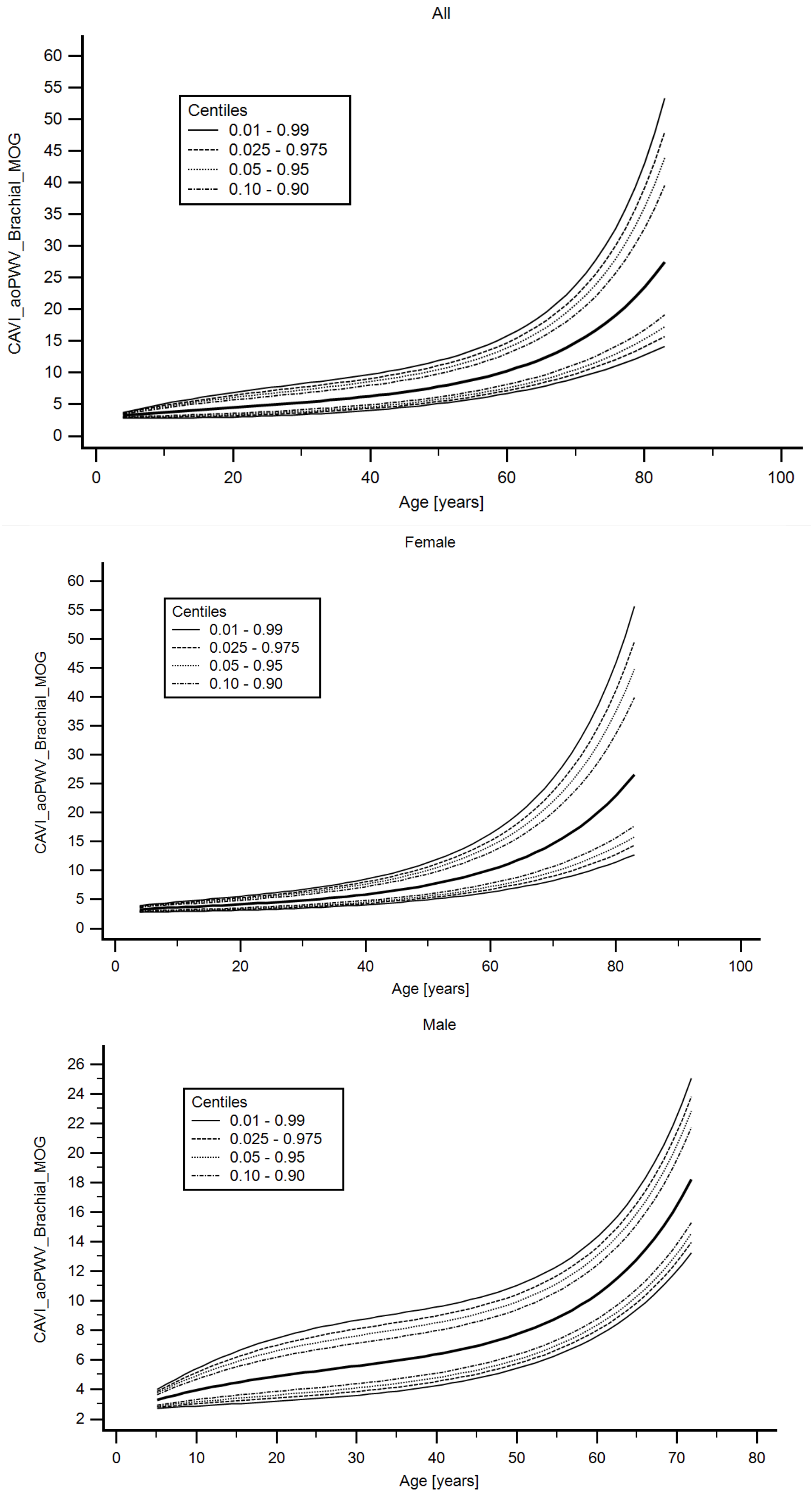

Figure S14. Age-related profiles for CAVI_aoPWV_Brachial_MOG, for subjects included in RIs group.

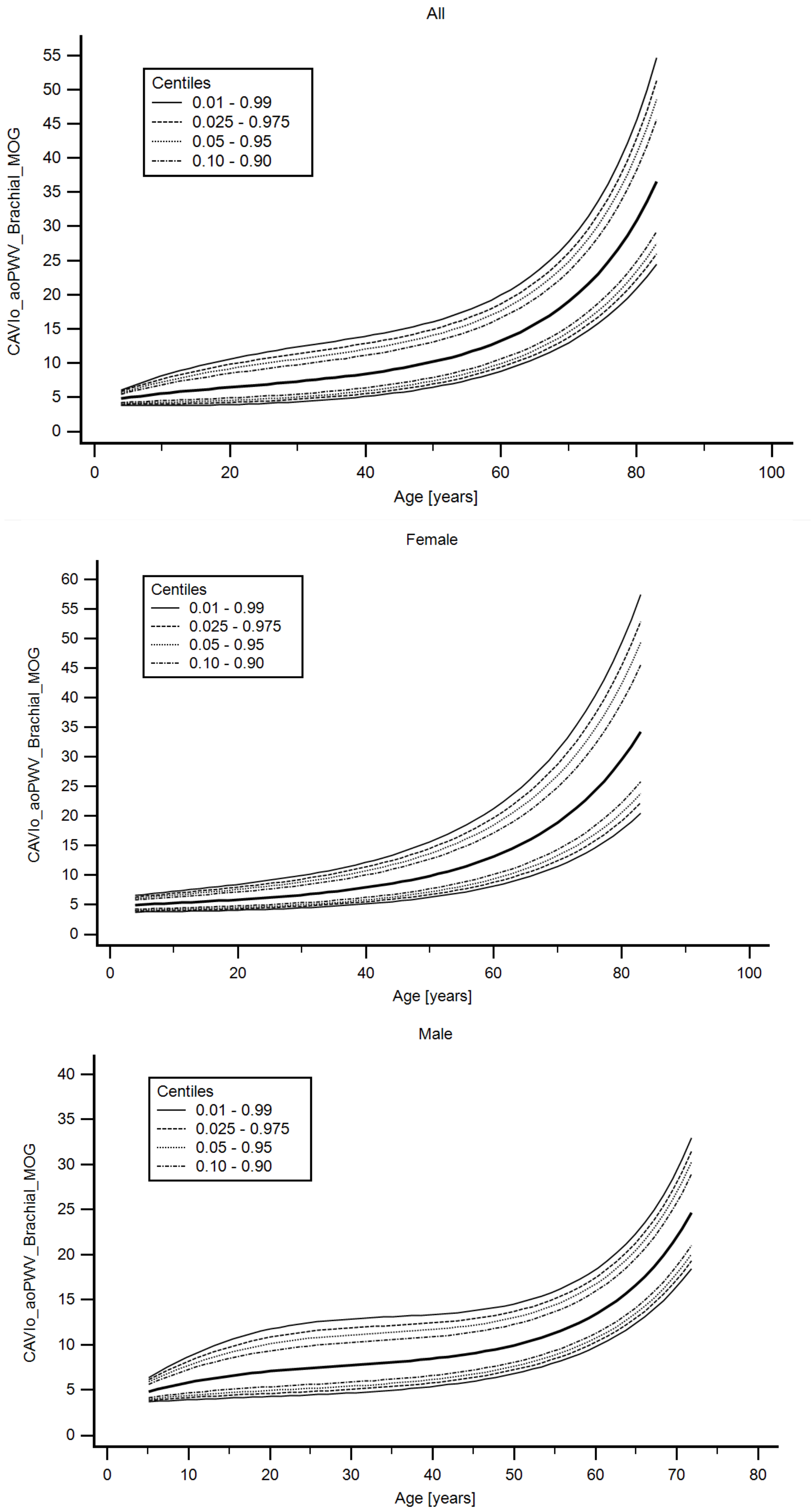

Figure S15. Age-related profiles for CAVIo_aoPWV_Brachial_MOG, for subjects included in RIs group.

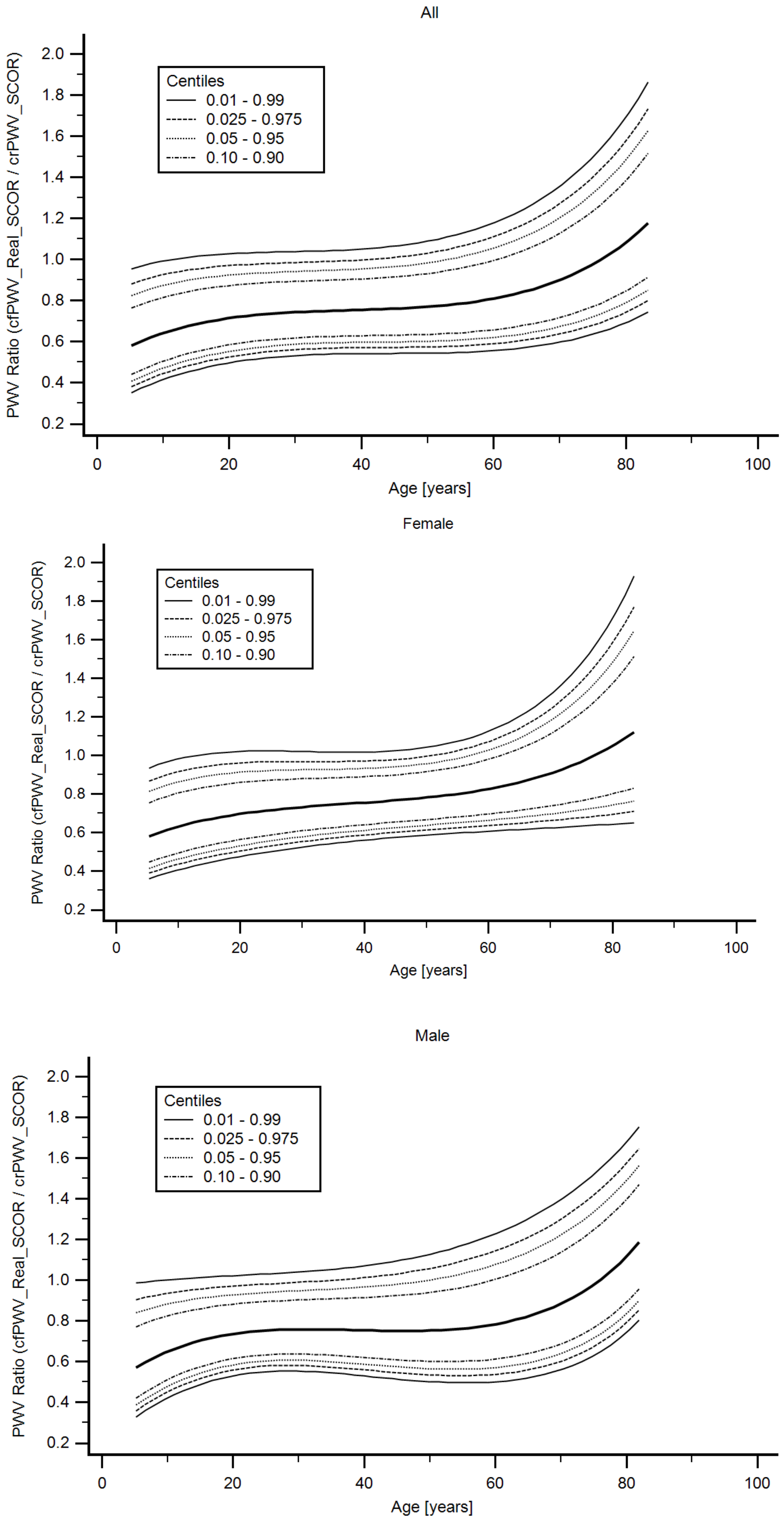

Figure S16. Age-related profiles for PWV_Ratio, for subjects included in RIs group.

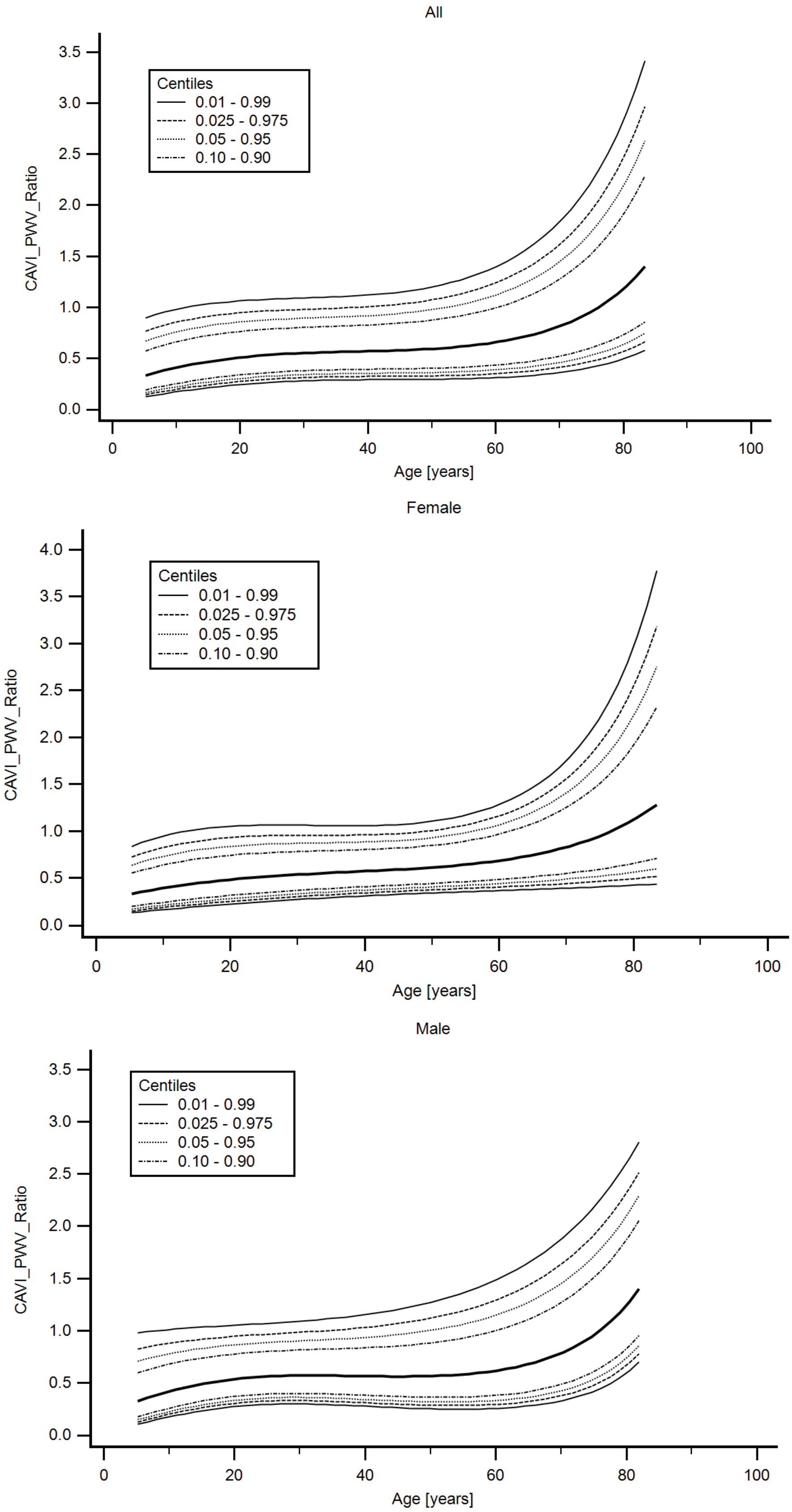

Figure S17. Age-related profiles for CAVI_PWV_Ratio, for subjects included in RIs group.

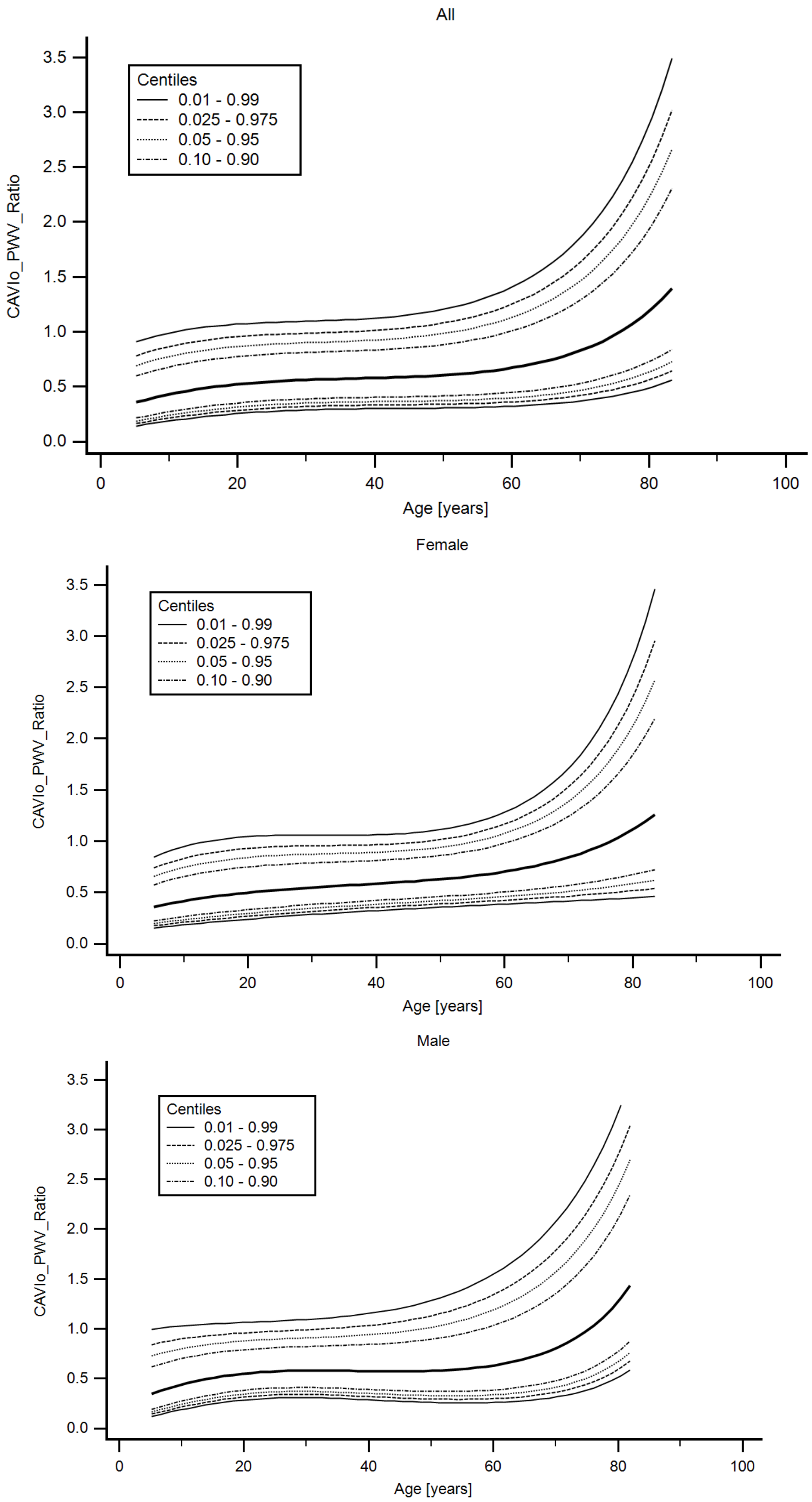

Figure S18. Age-related profiles for CAVIo_PWV_Ratio, for subjects included in RIs group.
